# Numerical simulation and investigation of soliton solutions and chaotic behavior to a stochastic nonlinear Schrödinger model with a random potential

**DOI:** 10.1371/journal.pone.0296678

**Published:** 2024-01-31

**Authors:** Asghar Ali, Jamshad Ahmad, Sara Javed, Rashida Hussain, Mohammed Kbiri Alaoui

**Affiliations:** 1 Department of Mathematics, Mirpur University of Science and Technology, (MUST) Mirpur-10250 (AJK), Mirpur, Pakistan; 2 Department of Mathematics, Faculty of Science, University of Gujrat, Gujrat, Pakistan; 3 Department of Mathematics, College of Science, King Khalid University, Abha, Saudi Arabia; Institute of Space Technology, PAKISTAN

## Abstract

The stochastic nonlinear Schrödinger model (SNLSM) in (1+1)-dimension with random potential is examined in this paper. The analysis of the evolution of nonlinear dispersive waves in a totally disordered medium depends heavily on the model under investigation. This study has three main objectives. Firstly, for the SNLSM, derive stochastic precise solutions by using the modified Sardar sub-equation technique. This technique is efficient and intuitive for solving such models, as shown by the generated solutions, which can be described as trigonometric, hyperbolic, bright, single and dark. Secondly, for obtaining numerical solutions to the SNLSM, the algorithms described here offer an accurate and efficient technique. Lastly, investigate the phase plane analysis of the perturbed and unperturbed dynamical system and the time series analysis of the governing model. The results show that the numerical and analytical techniques can be extended to solve other nonlinear partial differential equations in physics and engineering. The results of this study have a significant impact on how well we comprehend how solitons behave in physical systems. Additionally, they may serve as a foundation for the development of improved numerical techniques for handling challenging nonlinear partial differential equations.

## Introduction

Nonlinear partial differential equations (NLPDEs) have become essential tools for understanding the complex nonlinear physical phenomena that appear in a wide variety of models in a wide range of domains. NLPDEs are crucial in characterizing numerous scientific disciplines, such as ocean engineering, physics, geochemistry, fluid mechanics, geophysics, plasma physics, optical fibers and more capacity to capture and describe the complicated behaviors of real objects and dynamic processes. Due to its crucial function in revealing the fundamental properties of systems, the field of nonlinear phenomena is one of the most fascinating for analysts in today’s vanguard of scientific inquiry. As a result, the hunt for precise or analytical remedies for NLPDEs has developed into an exciting research area, arousing curiosity and inspiring novel studies [1–3]. Researchers have recently placed a great deal of emphasis on the pursuit of accurate solutions through the use of effective computing tools that streamline complex algebraic computations. The search for exact solutions has a specific position in mathematical physics, notably in the context of wave theory. Researchers work to provide accurate answers using cutting-edge computational methods that provide comprehensive insights into the behavior and characteristics of waves, enabling a deeper comprehension of the complex dynamics and phenomena seen in diverse physical systems. This focus on exact answers advances our theoretical understanding while also having tangible effects on areas like signal processing, communication and wave-based technologies [4–7].

The physical structures are better supported by these solutions of NLPDEs. In order to obtain the accurate solution for nonlinear physical models, various strong and effective methods, were developed and these methods are modified F-expansion technique [8, 9], the $\left(\frac{G^{\prime }}{G^{2}}\right)$-expansion function technique [10, 11], the first integral technique [12, 13], the Hirota bilinear transformation technique [14–16], the kather technique [17], the sin-Gordon expansion technique [18], the sin-cosine technique [19] and several others [20, 21]. Recently, other strategies are also taken into consideration for a range of lump solutions [22].

A powerful technique for resolving NLPDEs is numerical simulation [23]. This computational approach offers a useful and effective way to investigate and assess the intricate behavior of nonlinear phenomena across a range of scientific fields. Numerous benefits come from using numerical simulation to solve NLPDEs. It facilitates the investigation of various scenarios and parameter modifications by enabling the examination of a broad range of initial and boundary circumstances. Additionally, it offers a way to examine intricate systems for which there are no analytical solutions, providing insightful information that might direct subsequent theoretical and experimental research [24, 25].

Our main purpose is to find some new stochastic soliton solutions for the nonlinear Schrödinger model in (1+1)-dimension with random potential using modified Sardar sub-equation (MSSE) technique [26] and for numerical simulations using a modified variational iteration (VI) technique [27].

The SNLSM is a mathematical framework for describing the behavior of nonlinear systems in the presence of random fluctuations. It combines the ideas of quantum mechanics and stochastic processes. By including stochastic terms, which express the innate uncertainty and randomness observed in many physical systems, it expands the classical nonlinear Schrödinger model [28]. A modified Schrödinger model with additional variables that indicate stochastic influences govern the evolution of the wave function or wave envelope in the SNLSM [29]. These stochastic variables might originate from a variety of factors, including environmental interactions, random external forces and thermal variations [30].

The SNLSM, which has a stochastic soliton solution, serves as the primary representative model for understanding wave behavior in a variety of nonlinear applications, such as nonlinear optics, economics, biology and plasma physics [31]. In quantum optics, SNLSM has been used to analyze how measurement results affect the system state [32]. Additionally, the inhomogeneity of the medium in which the wave propagates the source of the noise can be explained using the noise term in Eq (1). Earlier the governing model has been studied for using the analytical techniques, now in this current research it has been done in a new way by using the novel analytical and numerical techniques to explore the novel results. Consider an SNLSM is [33]

$$\begin{array}{c} idR=[\Delta R+{L|R|}^{2}R]dt+\beta RdM, \end{array}$$ (1)

where R(x,t) is complex function of stochastic type, *β* represent noise intensity, M represent the brownian motion at one variable that is *t*, $M_{t}$ represent multiplicative noise and L represent nonlinear dispersive term. The novelty of the work is to explore the stochastic novel solution that is not explored in the literature along with studying the dynamical behavior of a perturbed and non-perturbed dynamical system, also compare the analytical and numerical results.

The layout of the work is structured as follows. The mathematical analysis describes the modified Sardar sub-equation technique. The next section covers applying the governing model that is considered in Eq (1). The phase plane analysis and time series analysis are provided. Results and discussions are provided in the next section. The study’s conclusion and future work are found in the last.

## Mathematical analysis

The modified Sardar sub-equation (MSSE) technique extends the capabilities of the original Sardar sub-equation technique by including more terms and instances in the ansatz for the solution, allowing it to solve a wider range of nonlinear equations. The method has been successfully applied to resolve NLPDEs in physics and mathematics multiple times.

Considering NLPDEs in their generic form

$$\begin{array}{c} G(v,v_{t},v_{x},v_{xx},v_{xt},\ldots )=0, \end{array}$$ (2)

where *v* = *v*(*x*, *t*) is a complex valued function, *x* represents space and *t* represents time.

**Step 1**. Assume the transformation of waves are

$$\begin{array}{c} R\left(x,t\right)=N\left(\xi \right)e^{i\alpha +\beta M\left(t\right)-\frac{1}{2}\beta^{2}t},\;\xi =U\left(x-pt\right),\;\alpha =-\sigma x+\gamma t+\theta_{0}, \end{array}$$ (3)

where *γ* represent frequency, *σ* represent wave number, *θ*_0_ represent phase angle, *β* represent noise intensity, U represent width and *p* represent velocity of solutions.

Putting the Eq (3) into the Eq (2), the resulting is a nonlinear ODE as:

$$\begin{array}{c} G(N\left(\xi \right),N^{\prime }\left(\xi \right),N^{\prime \prime }\left(\xi \right),\ldots )=0. \end{array}$$ (4)

**Step 2**. The given form describes the general solution of Eq (4), as per the method.

$$\begin{array}{c} N\left(\xi \right)=G_{0}+\sum_{j=1}^{J}G_{j}Q^{j}\left(\xi \right),\;G_{j}\neq \;0, \end{array}$$ (5)

where N=N(ξ) assures

$$\begin{array}{c} Q^{\prime }{\left(\xi \right)}^{2}=h_{2}Q{\left(\xi \right)}^{4}+h_{1}Q{\left(\xi \right)}^{2}+h_{0}, \end{array}$$ (6)

where *h*_0_ ≠ 1, *h*_1_ and *h*_2_ ≠ 0 are integers. Calculating the constants *G*_0_ and *G*_1_ and additionally, it is invertible for *G*_*j*_ to be zero. Determined the value of *J* using the balance principle. Following are the cases to Eq (6).

**Case-1**:

• If *h*_0_ = 0, *h*_1_ > 0 and *h*_2_ ≠ 0, then

$$\begin{array}{c} Q_{1}\left(\xi \right)=\sqrt{-\frac{h_{1}}{h_{2}}}\;\text{sech}\;(\sqrt{h_{1}}\left(\xi +\tau \right)). \end{array}$$ (7)

• If *h*_0_ = 0, *h*_1_ > 0 and *h*_2_ ≠ 0, then

$$\begin{array}{c} Q_{2}\left(\xi \right)=\sqrt{-\frac{h_{1}}{h_{2}}}\;\text{csch}\;(\sqrt{h_{1}}\left(\xi +\tau \right)). \end{array}$$ (8)

**Case-2**:

• For constants *k*_1_ and *k*_2_, let *h*_0_ = 0, *h*_1_ > 0 and *h*_2_ = + 4*k*_1_*k*_2_, then

$$\begin{array}{c} Q_{3}\left(\xi \right)=\frac{4k_{1}\sqrt{h_{1}}}{\left(4k_{1}^{2}-h_{2})\;\text{sinh}\;(\sqrt{h_{1}}\left(\xi +\tau \right))+(4k_{1}^{2}-h_{2})\;\text{cosh}\;(\sqrt{h_{1}}\left(\xi +\tau \right)\right)}. \end{array}$$ (9)

**Case-3**:

• For constants *E*_1_ and *E*_2_, let $h_{0}=\frac{h_{1}^{2}}{4h_{2}},h_{1}<0\;\text{and}\;h_{2}>0$, then

$$\begin{array}{c} Q_{4}\left(\xi \right)=\sqrt{-\frac{h_{1}}{2h_{2}}}\;\text{tanh}\;(\sqrt{-\frac{h_{1}}{2}}\left(\xi +\tau \right)). \end{array}$$ (10)

• For constants *E*_1_ and *E*_2_, let $h_{0}=\frac{h_{1}^{2}}{4h_{2}},h_{1}<0\;\text{and}\;h_{2}>0$, then

$$\begin{array}{c} Q_{5}\left(\xi \right)=\sqrt{-\frac{h_{1}}{2h_{2}}}\;\text{coth}\;(\sqrt{-\frac{h_{1}}{2}}\left(\xi +\tau \right)). \end{array}$$ (11)

• For constants *E*_1_ and *E*_2_, let $h_{0}=\frac{h_{1}^{2}}{4h_{2}},\;h_{1}<0\;\text{and}\;h_{2}>0$, then

$$\begin{array}{c} Q_{6}\left(\xi \right)=\sqrt{-\frac{h_{1}}{2h_{2}}}(\text{tanh}(\sqrt{-\frac{h_{1}}{2}}\left(\xi +\tau \right))+i\text{sech}(\sqrt{-2h_{1}}\left(\xi +\tau \right))). \end{array}$$ (12)

• For constants *E*_1_ and *E*_2_, let $h_{0}=\frac{h_{1}^{2}}{4h_{2}},h_{1}<0\;\text{and}\;h_{2}>0$, then

$$\begin{array}{c} Q_{7}\left(\xi \right)=\sqrt{-\frac{h_{1}}{8h_{2}}}(\text{tanh}(\sqrt{-\frac{h_{1}}{8}}\left(\xi +\tau \right))+\text{coth}(\sqrt{-\frac{h_{1}}{8}}\left(\xi +\tau \right))). \end{array}$$ (13)

• For constants *E*_1_ and *E*_2_, let $h_{0}=\frac{h_{1}^{2}}{4h_{2}},\;h_{1}<0\;\text{and}\;h_{2}>0$, then

$$\begin{array}{c} Q_{8}\left(\xi \right)=\frac{\sqrt{-\frac{h_{1}}{2h_{2}}}\;\text{cosh}\;(\sqrt{-2h_{1}}\left(\xi +\tau \right))}{\text{sinh}\;(\sqrt{-2h_{1}}\left(\xi +\tau \right))+i}. \end{array}$$ (14)

**Case-4**:

• Let *h*_0_ = 0, *h*_1_ < 0 and *h*_2_ ≠ 0, then

$$\begin{array}{c} Q_{9}\left(\xi \right)=\sqrt{-\frac{h_{1}}{h_{2}}}\;\text{sec}\;(\sqrt{-h_{1}}\left(\xi +\tau \right)). \end{array}$$ (15)

• Let *h*_0_ = 0, *h*_1_ < 0 and *h*_2_ ≠ 0, then

$$\begin{array}{c} Q_{10}\left(\xi \right)=\sqrt{-\frac{h_{1}}{h_{2}}}\;\text{csc}\;(\sqrt{-h_{1}}\left(\xi +\tau \right)). \end{array}$$ (16)

**Case-5**:

• Let $h_{0}=\frac{h_{1}^{2}}{4h_{2}},\;h_{1}>0$ and *h*_2_ > 0 and $E_{1}^{2}-E_{2}^{2}>0$, then

$$\begin{array}{c} Q_{11}\left(\xi \right)=\sqrt{-\frac{h_{1}}{2h_{2}}}\;\text{tan}\;(\sqrt{\frac{h_{1}}{2}}\left(\xi +\tau \right)). \end{array}$$ (17)

• Let $h_{0}=\frac{h_{1}^{2}}{4h_{2}},\;h_{1}>0$ and *h*_2_ > 0 and $R_{1}^{2}-R_{2}^{2}>0$, then

$$\begin{array}{c} Q_{12}\left(\xi \right)=-\sqrt{-\frac{h_{1}}{2h_{2}}}\;\text{cot}\;(\sqrt{\frac{h_{1}}{2}}\left(\xi +\tau \right)). \end{array}$$ (18)

• Let $h_{0}=\frac{h_{1}^{2}}{4h_{2}},h_{1}>0$ and *h*_2_ > 0 and $E_{1}^{2}-E_{2}^{2}>0$, then

$$\begin{array}{c} Q_{13}\left(\xi \right)=-\sqrt{-\frac{h_{1}}{2h_{2}}}(\text{tan}(\sqrt{2h_{1}}\left(\xi +\tau \right))-\text{sec}(\sqrt{2h_{1}}\left(\xi +\tau \right))). \end{array}$$ (19)

• Let $h_{0}=\frac{h_{1}^{2}}{4h_{2}},\;h_{1}>0$ and *h*_2_ > 0 and $E_{1}^{2}-E_{2}^{2}>0$, then

$$\begin{array}{c} Q_{14}\left(\xi \right)=\sqrt{-\frac{h_{1}}{8h_{2}}}(\text{tan}(\sqrt{\frac{h_{1}}{8}}\left(\xi +\tau \right))-\text{cot}(\sqrt{\frac{h_{1}}{8}}\left(\xi +\tau \right))). \end{array}$$ (20)

• Let $h_{0}=\frac{h_{1}^{2}}{4h_{2}},\;h_{1}>0$ and *h*_2_ > 0 and $E_{1}^{2}-E_{2}^{2}>0$, then

$$\begin{array}{c} Q_{15}\left(\xi \right)=\frac{\sqrt{-\frac{h_{1}}{2h_{2}}}(\sqrt{E_{1}^{2}-E_{2}^{2}}-S_{1}\;\text{cos}\;(\sqrt{2h_{1}}\left(\xi +\tau \right)))}{E_{2}+S_{1}\;\text{sin}\;(\sqrt{2h_{1}}\left(\xi +\tau \right))}, \end{array}$$ (21)

$$\begin{array}{c} Q_{16}\left(\xi \right)=\frac{\sqrt{-\frac{h_{1}}{2h_{2}}}\;\text{cos}\;(\sqrt{2h_{1}}\left(\xi +\tau \right))}{\text{sin}(\sqrt{2h_{1}}\left(\xi +\tau \right))-1}. \end{array}$$ (22)

**Case-6**:

• Let *h*_0_ = 0, *h*_1_ > 0, then

$$\begin{array}{c} Q_{17}\left(\xi \right)=\frac{4h_{1}e^{\sqrt{h_{1}}\left(\xi +\tau \right)}}{e^{2\sqrt{h_{1}}\left(\xi +\tau \right)}-4h_{1}h_{2}}. \end{array}$$ (23)

• Let *h*_0_ = 0, *h*_1_ > 0, then

$$\begin{array}{c} Q_{18}\left(\xi \right)=\frac{4h_{1}e^{\sqrt{h_{1}}\left(\xi +\tau \right)}}{1-4h_{1}h_{2}e^{2\sqrt{h_{1}}\left(\xi +\tau \right)}}. \end{array}$$ (24)

**Case-7**:

• Let *h*_0_ = 0, *h*_1_ = 0 and *h*_2_ > 0, then

$$\begin{array}{c} Q_{19}\left(\xi \right)=\frac{1}{\sqrt{h_{2}}\left(\xi +\tau \right)}. \end{array}$$ (25)

• Let *h*_0_ = 0, *h*_1_ = 0 & *h*_2_ > 0, then

$$\begin{array}{c} Q_{20}\left(\xi \right)=\frac{i}{\sqrt{h_{2}}\left(\xi +\tau \right)}. \end{array}$$ (26)

**Step 3**. By combining Eq (5) and its second-order necessary derivatives with Eqs (4) and (6) the resulting polynomial is a power of Q(ξ).

**Step 4**. The algebraic system of the equation was generated for *G*_0_, *G*_*n*_ (where *n* = 1, 2, 3, …) by gathering all the coefficients of the Q(ξ) that have the same power and further equating each coefficient to zero.

**Step 5**. At last, use Wolfram Mathematica to solve the algebraic systems of equations and determine the parameter values. We can solve Eq (1) by plugging these parameter values into Eq (5).

The (1+1)-dimensional SNLSM with random potential, for example, can be solved precisely using the MSSE technique. The method requires making an assumption about the answer in terms of extra variables and a singular function, then solving an algebraic system of equations to obtain the unknown constants.

## Applications

To generate a precise solution, let’s assume Eq (1). When inserting the stochastic wave’s Eq (3) into Eq (1), we obtain the nonlinear ordinary differential equation, which has imaginary and real parts, respectively.

$$\begin{array}{c} p=\gamma \sigma , \end{array}$$ (27)

$$\begin{array}{c} N^{\prime \prime }\left(\xi \right)+(\gamma -\sigma^{2})N\left(\xi \right)+LN{\left(\xi \right)}^{3}=0, \end{array}$$ (28)

where N(ξ) is stochastic function of complex-valued. From Eq (28), the homogeneous balancing term is

$$\begin{array}{c} N\left(\xi \right)=G_{1}Q\left(\xi \right)+G_{0}, \end{array}$$ (29)

where *G*_0_ and *G*_1_ are constant to be evaluated. Inserting Eq (29), and required derivatives into Eq (28). Then solve the system of equations to get the solution set.

• Set-1:

$$\begin{array}{c} \{G_{0}\to 0,G_{1}\to -\frac{i\sqrt{2}\sqrt{h_{2}}}{\sqrt{L}},\sigma \to -\sqrt{\gamma +h_{1}}\}. \end{array}$$ (30)

Using set-1 in Eq (30), and cases of Eqs (7)–(26) to obtain the required solutions.

$$\begin{array}{c} \begin{array}{c} R_{1,1}=-\frac{i\sqrt{2}\sqrt{-\frac{h_{1}}{h_{2}}}\sqrt{h_{2}}\;\text{exp}\;(i(x\sqrt{\gamma +h_{1}}+\gamma t+\vartheta )-\frac{\beta^{2}t}{2}+2\beta t)}{\sqrt{L}}\\ \times \frac{\text{sech}(\sqrt{h_{1}}\left(U\left(x-pt\right)+\tau \right))}{\sqrt{L}}, \end{array} \end{array}$$ (31)

$$\begin{array}{c} \begin{array}{c} R_{1,2}=-\frac{i\sqrt{2}\sqrt{-\frac{h_{1}}{h_{2}}}\sqrt{h_{2}}\;\text{exp}\;(i(x\sqrt{\gamma +h_{1}}+\gamma t+\vartheta )-\frac{\beta^{2}t}{2}+2\beta t)}{\sqrt{L}}\\ \times \frac{\text{csch}(\sqrt{h_{1}}\left(U\left(x-pt\right)+\tau \right))}{\sqrt{L}}, \end{array} \end{array}$$ (32)

$$\begin{array}{c} \begin{array}{c} R_{1,3}=-\frac{4i\sqrt{2}\sqrt{h_{1}}\sqrt{h_{2}}k_{1}\;\text{exp}\;(i(x\sqrt{\gamma +h_{1}}+\gamma t+\vartheta )-\frac{\beta^{2}t}{2}+2\beta t)}{\sqrt{L}(\text{sinh}\;(\sqrt{h_{1}}\left(U\left(x-pt\right)+\tau \right))+\text{cosh}\;(\sqrt{h_{1}}\left(U\left(x-pt\right)+\tau \right)))}\\ \times \frac{1}{\left(4k_{1}^{2}-h_{2}\right)}, \end{array} \end{array}$$ (33)

$$\begin{array}{c} R_{1,4}=-\frac{i\sqrt{-\frac{h_{1}}{h_{2}}}\sqrt{h_{2}}\;\text{exp}\;(i(x\sqrt{\gamma +h_{1}}+\gamma t+\vartheta )-\frac{\beta^{2}t}{2}+2\beta t)\;\text{tanh}\;(\frac{\sqrt{-h_{1}}\left(U\left(x-pt\right)+\tau \right)}{\sqrt{2}})}{\sqrt{L}}, \end{array}$$ (34)

$$\begin{array}{c} R_{1,5}=-\frac{i\sqrt{-\frac{h_{1}}{h_{2}}}\sqrt{h_{2}}\;\text{exp}\;(i(x\sqrt{\gamma +h_{1}}+\gamma t+\vartheta )-\frac{\beta^{2}t}{2}+2\beta t)\;\text{coth}\;(\frac{\sqrt{-h_{1}}\left(U\left(x-pt\right)+\tau \right)}{\sqrt{2}})}{\sqrt{L}}, \end{array}$$ (35)

$$\begin{array}{c} \begin{array}{c} R_{1,6}=-\frac{i\sqrt{-\frac{h_{1}}{h_{2}}}\sqrt{h_{2}}\;\text{exp}\;(i(x\sqrt{\gamma +h_{1}}+\gamma t+\vartheta )-\frac{\beta^{2}t}{2}+2\beta t)(\text{tanh}\;(\frac{\sqrt{-h_{1}}\left(U\left(x-pt\right)+\tau \right)}{\sqrt{2}}))}{\sqrt{L}}\\ +\frac{+i\text{sech}\;(\sqrt{2}\sqrt{-h_{1}}\left(U\left(x-pt\right)+\tau \right))}{\sqrt{L}}, \end{array} \end{array}$$ (36)

$$\begin{array}{c} \begin{array}{c} R_{1,7}=-\frac{i\sqrt{-\frac{h_{1}}{h_{2}}}\sqrt{h_{2}}\;\text{exp}\;(i(x\sqrt{\gamma +h_{1}}+\gamma t+\vartheta )-\frac{\beta^{2}t}{2}+2\beta t)(\text{tanh}\;(\frac{\sqrt{-h_{1}}\left(U\left(x-pt\right)+\tau \right)}{2\sqrt{2}}))}{2\sqrt{L}}\\ +\frac{\text{coth}\;(\frac{\sqrt{-h_{1}}\left(U\left(x-pt\right)+\tau \right)}{2\sqrt{2}})}{2\sqrt{L}}, \end{array} \end{array}$$ (37)

$$\begin{array}{c} \begin{array}{c} R_{1,8}=-\frac{i\sqrt{-\frac{h_{1}}{h_{2}}}\sqrt{h_{2}}\;\text{exp}\;(i(x\sqrt{\gamma +h_{1}}+\gamma t+\vartheta )-\frac{\beta^{2}t}{2}+2\beta t)}{\sqrt{L}(\text{sinh}\;(\sqrt{2}\sqrt{-h_{1}}\left(U\left(x-pt\right)+\tau \right))+i)}\\ \;\times (\text{cosh}\;(\sqrt{2}\sqrt{-h_{1}}\left(U\left(x-pt\right)+\tau \right))), \end{array} \end{array}$$ (38)

$$\begin{array}{c} \begin{array}{c} R_{1,9}=-\frac{i\sqrt{2}\sqrt{-\frac{h_{1}}{h_{2}}}\sqrt{h_{2}}\;\text{exp}\;(i(x\sqrt{\gamma +h_{1}}+\gamma t+\vartheta )-\frac{\beta^{2}t}{2}+2\beta t)}{\sqrt{L}}\\ \times (\text{sec}\;(\sqrt{-h_{1}}\left(U\left(x-pt\right)+\tau \right))), \end{array} \end{array}$$ (39)

$$\begin{array}{c} \begin{array}{c} R_{1,10}=-\frac{i\sqrt{2}\sqrt{-\frac{h_{1}}{h_{2}}}\sqrt{h_{2}}\;\text{exp}\;(i(x\sqrt{\gamma +h_{1}}+\gamma t+\vartheta )-\frac{\beta^{2}t}{2}+2\beta t)}{\sqrt{L}}\\ \times (\text{csc}\;(\sqrt{-h_{1}}\left(U\left(x-pt\right)+\tau \right))), \end{array} \end{array}$$ (40)

$$\begin{array}{c} R_{1,11}=-\frac{i\sqrt{-\frac{h_{1}}{h_{2}}}\sqrt{h_{2}}\;\text{exp}\;(i(x\sqrt{\gamma +h_{1}}+\gamma t+\vartheta )-\frac{\beta^{2}t}{2}+2\beta t)\;\text{tan}\;(\frac{\sqrt{h_{1}}\left(U\left(x-pt\right)+\tau \right)}{\sqrt{2}})}{\sqrt{L}}, \end{array}$$ (41)

$$\begin{array}{c} R_{1,12}=\frac{i\sqrt{-\frac{h_{1}}{h_{2}}}\sqrt{h_{2}}\;\text{exp}\;(i(x\sqrt{\gamma +h_{1}}+\gamma t+\vartheta )-\frac{\beta^{2}t}{2}+2\beta t)\;\text{cot}\;(\frac{\sqrt{h_{1}}\left(U\left(x-pt\right)+\tau \right)}{\sqrt{2}})}{\sqrt{L}}, \end{array}$$ (42)

$$\begin{array}{c} \begin{array}{c} R_{1,13}=\frac{i\sqrt{-\frac{h_{1}}{h_{2}}}\sqrt{h_{2}}\;e^{\left(i(x\sqrt{\gamma +h_{1}}+\gamma t+\vartheta )-\frac{\beta^{2}t}{2}+2\beta t\right)}(\text{tan}(\sqrt{2}\sqrt{h_{1}}\left(U\left(x-pt\right)+\tau \right)))}{\sqrt{L}}\\ -\frac{\text{sec}(\sqrt{2}\sqrt{h_{1}}\left(U\left(x-pt\right)+\tau \right))}{\sqrt{L}}, \end{array} \end{array}$$ (43)

$$\begin{array}{c} \begin{array}{c} R_{1,14}=-\frac{i\sqrt{-\frac{h_{1}}{h_{2}}}\sqrt{h_{2}}\;\text{exp}\;(i(x\sqrt{\gamma +h_{1}}+\gamma t+\vartheta )-\frac{\beta^{2}t}{2}+2\beta t)(\text{tan}(\frac{\sqrt{h_{1}}\left(U\left(x-pt\right)+\tau \right)}{2\sqrt{2}}))}{2\sqrt{L}}\\ -\frac{\text{cot}(\frac{\sqrt{h_{1}}\left(U\left(x-pt\right)+\tau \right)}{2\sqrt{2}})}{2\sqrt{L}}, \end{array} \end{array}$$ (44)

$$\begin{array}{c} \begin{array}{c} R_{1,15}=-\frac{i\sqrt{-\frac{h_{1}}{h_{2}}}\sqrt{h_{2}}\;\text{exp}\;(i(x\sqrt{\gamma +h_{1}}+\gamma t+\vartheta )-\frac{\beta^{2}t}{2}+2\beta t)}{\sqrt{L}(S_{1}\;\text{sin}\;(\sqrt{2}\sqrt{h_{1}}\left(U\left(x-pt\right)+\tau \right))+e_{2})}\\ \times (\sqrt{e_{1}^{2}-e_{2}^{2}}-S_{1}\;\text{cos}\;(\sqrt{2}\sqrt{h_{1}}\left(U\left(x-pt\right)+\tau \right))), \end{array} \end{array}$$ (45)

$$\begin{array}{c} \begin{array}{c} R_{1,16}=-\frac{i\sqrt{-\frac{h_{1}}{h_{2}}}\sqrt{h_{2}}\;\text{exp}\;(i(x\sqrt{\gamma +h_{1}}+\gamma t+\vartheta )-\frac{\beta^{2}t}{2}+2\beta t)}{\sqrt{L}(\text{sin}(\sqrt{2}\sqrt{h_{1}}\left(U\left(x-pt\right)+\tau \right))-1)}\\ \;(\text{cos}(\sqrt{2}\sqrt{h_{1}}\left(U\left(x-pt\right)+\tau \right))), \end{array} \end{array}$$ (46)

$$\begin{array}{c} R_{1,17}=-\frac{4i\sqrt{2}h_{1}\sqrt{h_{2}}\;\text{exp}\;(\sqrt{h_{1}}\left(U\left(x-pt\right)+\tau \right)+i(x\sqrt{\gamma +h_{1}}+\gamma t+\vartheta )-\frac{\beta^{2}t}{2}+2\beta t)}{\sqrt{L}(e^{2\sqrt{h_{1}}\left(U\left(x-pt\right)+\tau \right)}-4h_{1}h_{2})}, \end{array}$$ (47)

$$\begin{array}{c} R_{1,18}=-\frac{4i\sqrt{2}h_{1}\sqrt{h_{2}}\;\text{exp}\;(\sqrt{h_{1}}\left(U\left(x-pt\right)+\tau \right)+i(x\sqrt{\gamma +h_{1}}+\gamma t+\vartheta )-\frac{\beta^{2}t}{2}+\beta t)}{\sqrt{L}(1-4h_{1}h_{2}e^{2\sqrt{h_{1}}\left(U\left(x-pt\right)+\tau \right)})}, \end{array}$$ (48)

$$\begin{array}{c} R_{1,19}=-\frac{i\sqrt{2}e^{i(x\sqrt{\gamma +h_{1}}+\gamma t+\vartheta )-\frac{\beta^{2}t}{2}+\beta t}}{\sqrt{L}\left(U\left(x-pt\right)+\tau \right)}, \end{array}$$ (49)

$$\begin{array}{c} R_{1,20}=\frac{\sqrt{2}e^{i(x\sqrt{\gamma +h_{1}}+\gamma t+\vartheta )-\frac{\beta^{2}t}{2}+\beta t}}{\sqrt{L}\left(U\left(x-pt\right)+\tau \right)}. \end{array}$$ (50)

### The modified variational iteration technique

We use the modified variational iteration technique to assess the accuracy of the analytical solutions we previously obtained [34]. The following equations provide the semi-analytical solutions for the model under investigation, which is represented by Eq (34).

$$\begin{array}{c} \begin{array}{c} R_{0}\left(x,t\right)=3-\text{tanh}(\frac{x}{2}),\\ R_{1}\left(x,t\right)=\frac{3}{8}t(\text{sinh}(\frac{3x}{4})-13\;\text{sinh}\;(\frac{x}{2})){\text{sech}}^{7}(\frac{x}{2})-\text{tanh}(\frac{x}{2})+7. \end{array} \end{array}$$ (51)

Novel stochastic solutions for the model under examination are found in this part using the modified VI technique. The parameter values are calculated using the following formats

$$\alpha \to -2\xi ,\gamma \to h^{2}.$$

construction of solitary wave solutions for Eq (1) is performed by

$$\begin{array}{c} R\left(x,t\right)=b_{0}+2h(1+\frac{1}{-1+\zeta e^{h(th^{2}+x+c_{1})}}), \end{array}$$ (52)

$$\begin{array}{c} R\left(x,t\right)=b_{0}-\frac{2\zeta h}{h℧e^{-h(th^{2}+x)}-\zeta }, \end{array}$$ (53)

$$\begin{array}{c} R\left(x,t\right)=b_{0}+h\;\text{tanh}(\frac{1}{2}h(th^{2}+x))+h, \end{array}$$ (54)

$$\begin{array}{c} R\left(x,t\right)=b_{0}+h\;\text{coth}(\frac{1}{2}h(th^{2}+x))+h, \end{array}$$ (55)

$$\begin{array}{c} R\left(x,t\right)=b_{0}+\frac{2h^{2}}{1-℧e^{-h(th^{2}+x)}}. \end{array}$$ (56)

We then use the modified variational iteration technique to assess the accuracy of the earlier-derived analytical answers. The model’s ensuing semi-analytical solutions, as given by Eq (54), are represented as follows

$$\begin{array}{c} R_{0}\left(x,t\right)=\text{tanh}(\frac{x}{2}), \end{array}$$ (57)

$$\begin{array}{c} R_{1}\left(x,t\right)=\frac{1}{2}\text{tanh}(\frac{x}{2})(3t\;{\text{sech}}^{4}(\frac{x}{2})+2), \end{array}$$ (58)

$$\begin{array}{c} R_{2}(x,t)=\frac{2}{3}t\;{\text{sech}}^{5}\left(\frac{x}{3}\right)\left(\frac{1.5}{{\left(\text{cosh}(x)+1\right)}^{4}}\left(-3\;\text{cosh}(x)\left(3652t^{2}\;\text{tanh}\left(\frac{x}{3}\right){\text{sech}}^{3}\left(\frac{2x}{3}\right)\right)\right)\right)\\ -3t(25t+7)\text{sinh}(3x)s+226t+5)+\text{cosh}(3x)(-25t\;\text{sinh}(12x)+31t-5)\\ +2t\left(t\;\text{tanh}(\frac{4x}{5})\left(-331t\;{\text{sech}}^{5}\left(\frac{3x}{5}\right)\right)\right)-7765t+8960)\\ +\text{sinh}(3x)\left(654t(76t-3){\text{sech}}^{2}\left(\frac{3x}{8}\right)-6743t-23\right)+543t-30\\ \left(+2\;\text{sinh}(5x)+\text{cosh}(7x)\left(\frac{74}{\text{cosh}(4x)+5}-9\right)\right)+6\;\text{tanh}\left(\frac{3x}{7}\right). \end{array}$$ (59)

The comparison of analytical stochastic solutions and numerical approximation solutions is given in the form of tables.

## Phase plane analysis

A significant method for examining the behavior of dynamical systems, both perturbed and unperturbed, is phase plane analysis. In phase plane analysis, we commonly use state variables and their derivatives to represent the dynamics of a system in a two-dimensional phase space. We can see the behavior of the system and analyze it thanks to this depiction. In some circumstances, the perturbation may also introduce new characteristics like chaotic behavior, limit cycles, or bifurcations [35]. Phase plane analysis tools, such as locating closed curves or areas where the trajectories are constrained, can be used to investigate these occurrences. It’s crucial to remember that the feasibility and precision of phase plane analysis can be impacted by the complexity of the perturbed system and the type of the perturbation term. To gain useful insights into the perturbed dynamical system, numerical approaches or more sophisticated techniques like averaging or perturbation theory may be necessary in particular circumstances [36].

Now, Eq (28) will be examined using the bifurcation theory and the phase portrait analysis technique [37]. A dynamical system that has a bifurcation as determined by Eq (28) will alter qualitatively due to changing parameters. Bifurcation theory offers a way to examine the bifurcations that occur within a family. This is accomplished by identifying frequent bifurcation patterns. The planer dynamical system of an unperturbed system for Eq (28) can be written as follows by using the Galilean transformation

$$\begin{array}{c} \begin{array}{c} \frac{dN}{d\xi }=L_{1},\\ \frac{d^{2}N}{d\xi^{2}}=L_{1}^{\prime }. \end{array} \end{array}$$ (60)

Insert the above transformation in Eq (28), we get

$$\begin{array}{c} L_{1}^{\prime }=-\left(\gamma -\sigma^{2}\right)N-LN^{3}, \end{array}$$

Thus, we obtained the required dynamical system for the unperturbed system

$$\begin{array}{c} \begin{array}{c} \frac{dN}{d\xi }=L_{1},\\ \frac{d^{2}N}{d\xi^{2}}=-\left(\sigma^{2}-\gamma \right)N-LN^{3}. \end{array} \end{array}$$ (61)

Assume the functions for F and W as:

$$\begin{array}{c} \begin{array}{c} F^{\prime }=F\left(F,W\right),\\ W^{\prime }=W\left(F,W\right). \end{array} \end{array}$$ (62)

As $W^{\prime }=T_{1}N-T_{2}N^{3}$, where *T*_1_ = *σ*^2^ − *γ* and $T_{2}=L$. So that $F^{\prime }=N^{\prime }=L_{1}$ and $W^{\prime }=N^{\prime \prime }=T_{1}N-T_{2}N^{3}$. That is

$$\begin{array}{c} J\left(F,W\right)=[\begin{array}{cc} \frac{\partial F}{\partial F} & \frac{\partial F}{\partial W}\\ \frac{\partial W}{\partial F} & \frac{\partial W}{\partial W} \end{array}], \end{array}$$

$$\begin{array}{c} \begin{array}{c} J\left(F,W\right)=|\begin{array}{cc} 0 & 1\\ T_{1}N-3LN^{2} & 0 \end{array}|=-T_{1}N+3T_{2}N^{2}. \end{array} \end{array}$$ (63)

where *T*_1_ and *T*_2_ are a real number. The equilibrium points of planar dynamical system (61) are given by

$$q_{1}=\left(0,0\right),\;q_{2}=(\sqrt{\frac{\sigma -\gamma }{L}},0),\;q_{3}=(-\sqrt{\frac{\sigma -\gamma }{L}},0).$$

Hence, (*F*, *W*) is the saddle point regarding J(F,W)<0, moreover, it would be the center point if J(F,W)=0 and it would be the cuspidal point if J(F,W)>0. The following results are observed to discuss the nature of a planar dynamical system of Eq (61) for the unperturbed system at equilibrium points.

***Case* (i)**: *T*_1_ < 0 **and** *T*_2_ > 0.

By using *γ* = −2, *σ* = 0.7 and L=2.7, the equilibrium points $q_{1}=\left(0,0\right),\;q_{2}=\left(\sqrt{1},0\right),\;q_{3}=\left(-\sqrt{1},0\right)$ have been retrieved from system (61) shown in Fig 16. It is observed, by the phase portrait, that *q*_2_ and *q*_3_ show cuspidal points, whereas *q*_1_ is a saddle point.

***Case* (ii)**: *T*_1_ > 0 **and** *T*_2_ > 0.

By using *γ* = 1.4, *σ* = 0.7 and L=0.7, there exists only one real equilibrium point *q*_1_ = (0, 0), two complex equilibrium points $q_{2}=\left(\sqrt{2}i,0\right)$ and $q_{3}=\left(-\sqrt{2}i,0\right)$, which has been obtained from system (61) and shown in Fig 17. It is observed that *q*_1_ and *q*_3_ are center points and *q*_2_ is a saddle point.

***Case* (iii)**: *T*_1_ < 0 **and** *T*_2_ < 0.

By using *γ* = 1.2, *σ* = −0.7 and L=0.7, there exists only one real equilibrium point *q*_1_ = (0, 0). It is observed that *q*_1_ is a center point and shown in Fig 18.

***Case* (iv)**: *T*_1_ > 0 **and** *T*_2_ < 0.

By using *γ* = 1.2, *σ* = −0.7 and L=0.7, there exists only one complex equilibrium point $q_{2}=\left(\sqrt{2}i,0\right)$. It is observed that *q*_2_ is a saddle point and shown in Fig 19.

When we apply the perturbation term to the dynamical system of Eq (61), where cos(*αϵ*) is the perturbation term, *α* is amplitude and *ϵ* is angle term in the perturbed dynamic system of following Eq (64), the resulting graphs as shown in Fig 9.

$$\begin{array}{c} \begin{array}{c} \frac{dN}{d\xi }=L_{1},\\ \frac{d^{2}N}{d\xi^{2}}=-\left(\sigma^{2}-\gamma \right)N-LN^{3}+\text{cos}\left(\alpha \epsilon \right). \end{array} \end{array}$$ (64)

To examine the behavior of dynamical systems, phase plane analysis can be used on both unperturbed and disturbed systems. We may learn a lot about the dynamics of the system, whether it is unperturbed or exposed to minor perturbations, by visualizing trajectories, examining critical points, and taking stability features into consideration.

### Time series analysis

An effective method for examining the characteristics and behavior of dynamical systems based on temporally dependent data is time series analysis. In order to understand and evaluate the underlying dynamical system, it seeks to extract meaningful facts, patterns, and correlations from the time series data [38]. It’s essential to note that time series analysis makes the assumption that the data observed is a realization of a stochastic process and that the models found are approximations of the genuine underlying dynamics. The suitability of the selected model, the quality and representativeness of the data, and the assumptions made throughout the study all affect how accurate and reliable the analysis is.

## Results and discussions

The originality of the present study is highlighted in this section by a thorough comparison of the evaluated results with the previously computed outcomes. In [39], Huang D, discusses the new iterative technique and the fractional power series technique as approximate solutions to the time-fractional Fokker-Planck model (TFFPM). This study, used (1+1)-dimensional stochastic nonlinear Schrödinger model with random potential and obtain the stochastic novel solitons solutions by using the analytical technique of the MSSE technique and for numerical approximation stochastic solution used modified VI technique. The solutions are novel and have not been investigated before. Such stochastic solitons are displayed in Eqs (31)–(50).

From Eq (31), $R_{1,1}$ is bright stochastic soliton. From Eq (32), $R_{1,2}$ is singular stochastic soliton. From Eq (33), $R_{1,3}$ is hyperbolic solution. Eq (34) represent the dark stochastic soliton of $R_{1,4}$. Eq (35) represent the singular stochastic soliton of $R_{1,5}$. Eq (36) represents the combo dark and bright stochastic soliton of $R_{1,6}$.

Eq (37) represents the combo of singular and dark stochastic soliton of $R_{1,7}$. Eq (38) represents the hyperbolic stochastic soliton of $R_{1,8}$. Eqs (39)–(46) represents the trigonometric stochastic soliton of $R_{1,9}-R_{1,16}$. Eqs (47) and (48) represents the exponential stochastic soliton of $R_{1,17}\text{and}R_{1,18}$. Eqs (49) and (50) represents the plane wave stochastic soliton of $R_{1,19}\text{and}R_{1,20}$.

The phase plane analysis results are qualitative in nature, revealing details on the qualitative traits and inclinations of the system’s dynamics. They enable us to recognize the system’s chaotic behavior, limit cycles and attractors behavior, limit cycles, and attractors in the system. Time series research frequently yields quantitative conclusions that let us base our forecasts and predictions on observable data and approximated models.

### Graphical illustration

**Fig 1:**
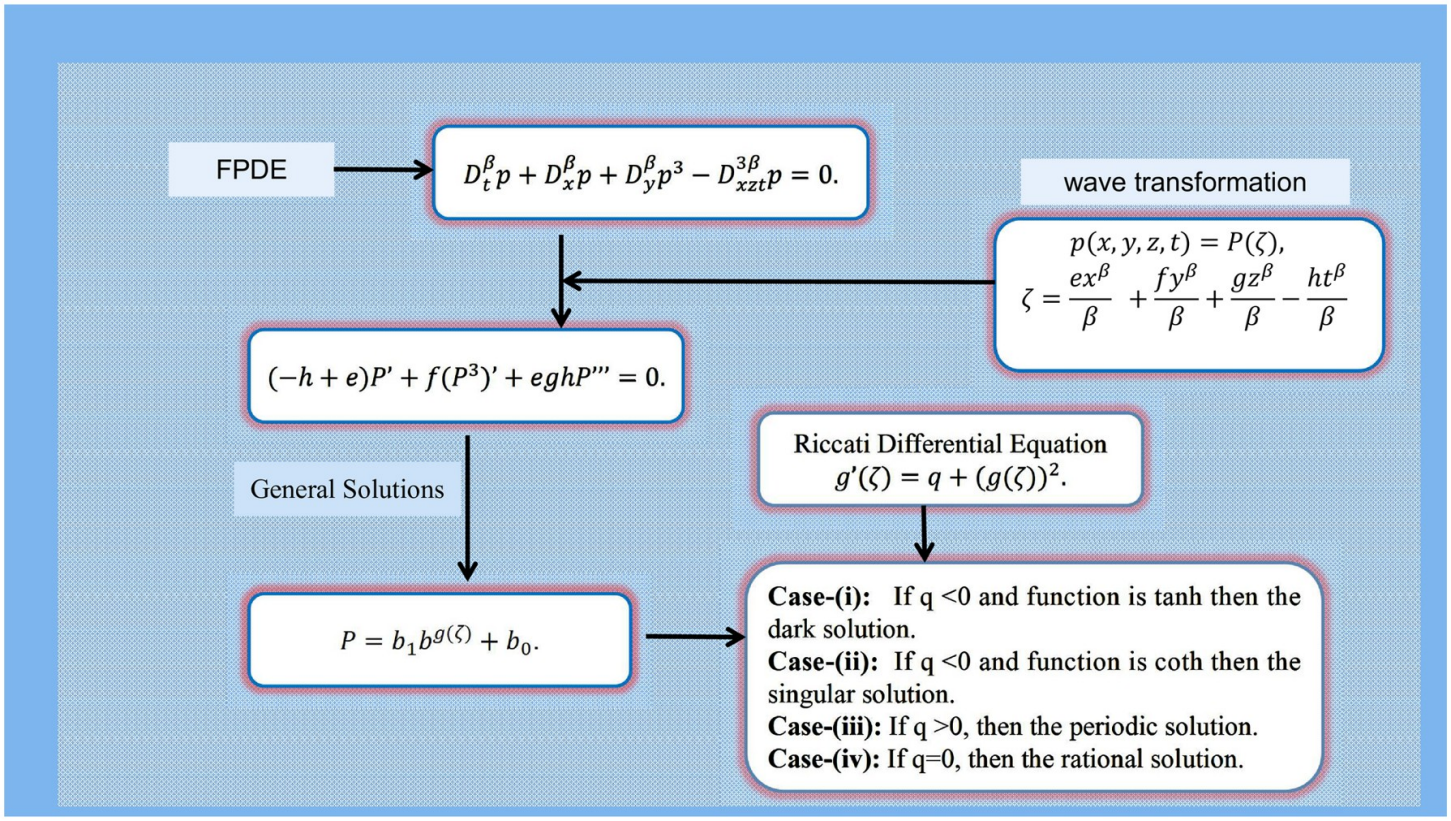
Flow chart of using analytical technique.

**Fig 2:**
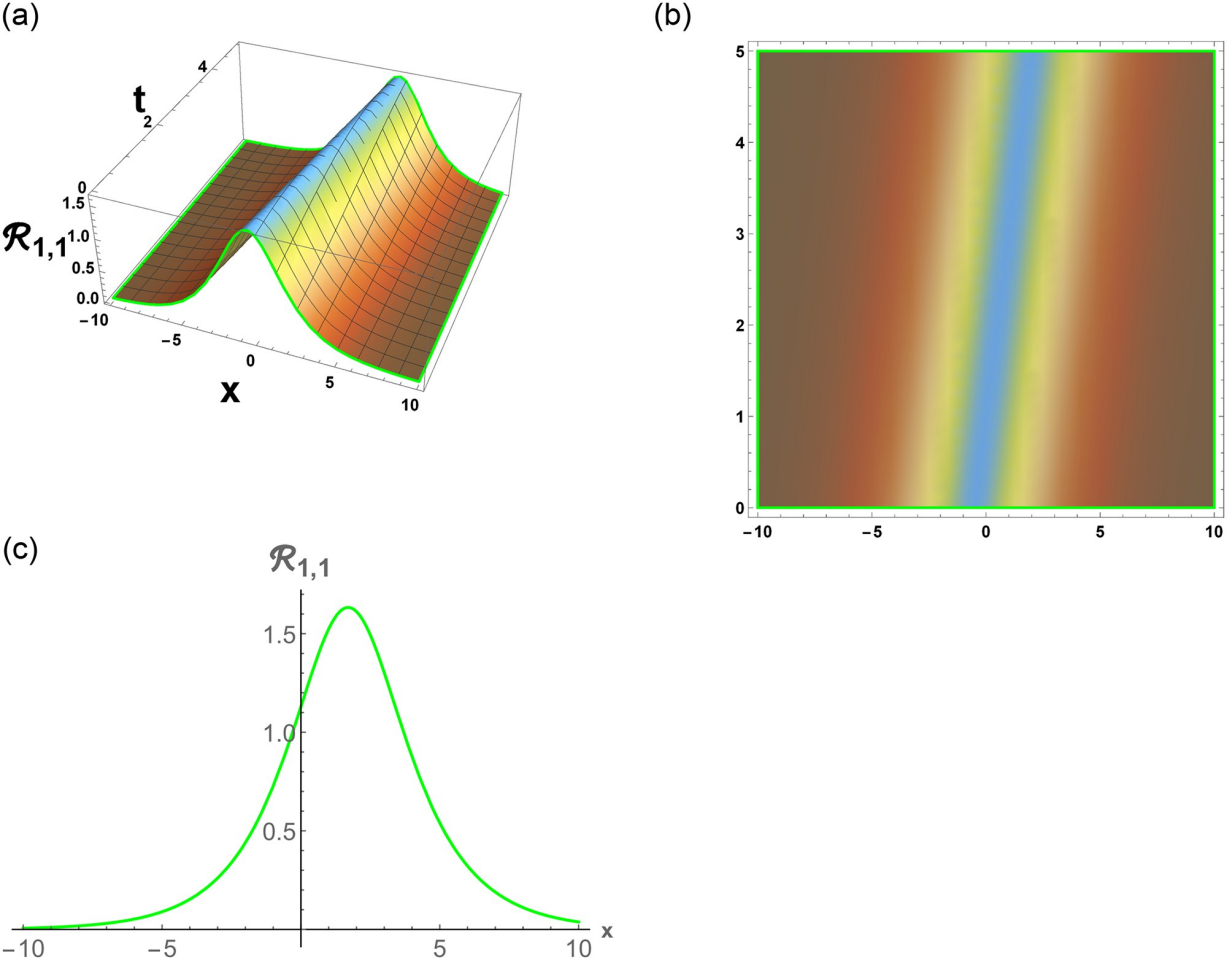
Physical depiction of $R_{1,1}$ under parametric values are $h_{1}=0.2,\;h_{2}=1.2,\;\gamma =0.3,\;\theta =0.1,\;\beta =0.6,\;U=1.2,\;p=1.1\;\text{and}\;\tau =0.3$. (a) 3-D, (b) Density, (c) 2-D.

**Fig 3:**
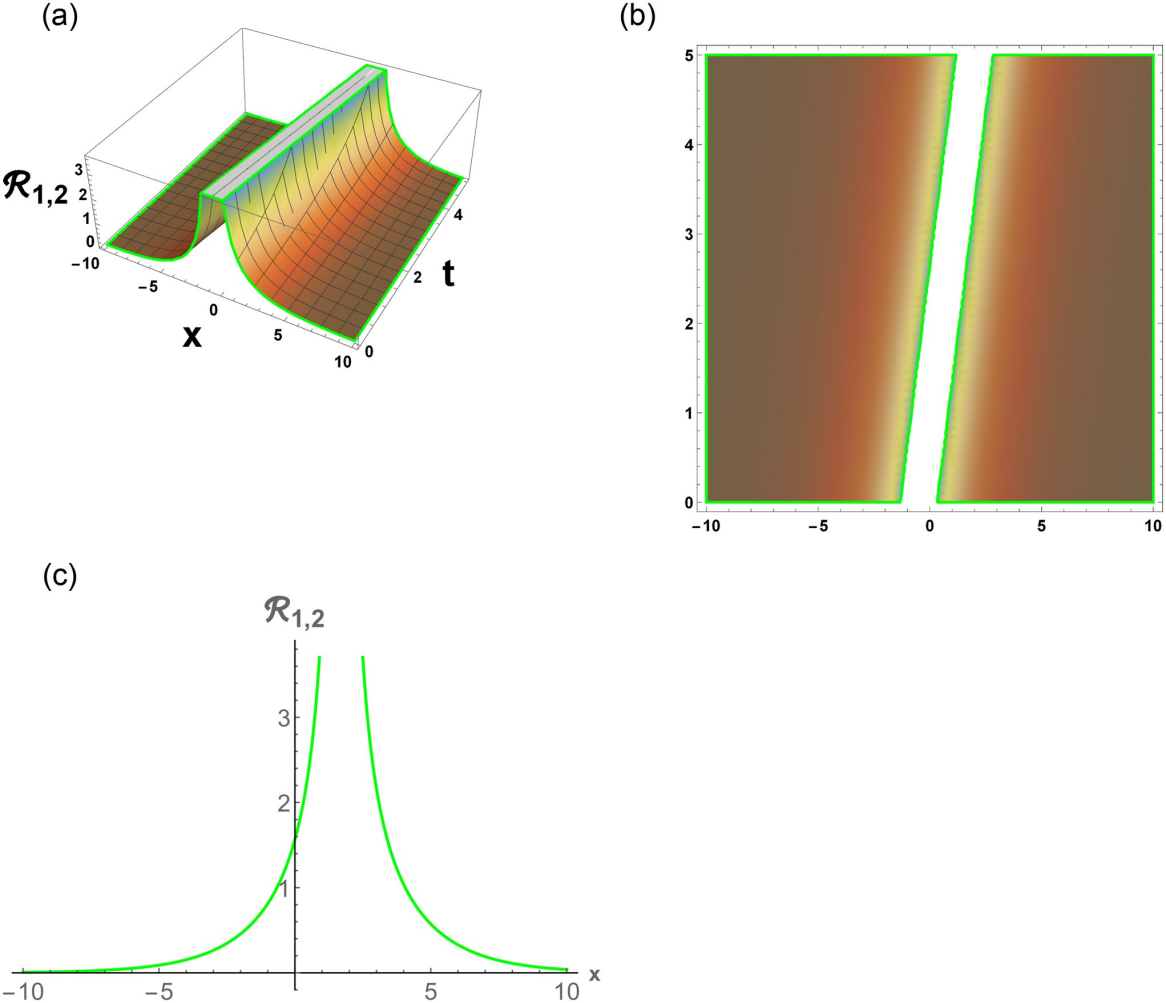
Physical depiction of $R_{1,2}$ under parametric values are $h_{1}=0.02,\;h_{2}=1.3,\;\gamma =0.1,\;\theta =0.21,\;\beta =0.6,\;U=1.2,\;p=1.1\;\text{and}\;\tau =0.23$. (a) 3-D, (b) Density, (c) 2-D.

**Fig 4:**
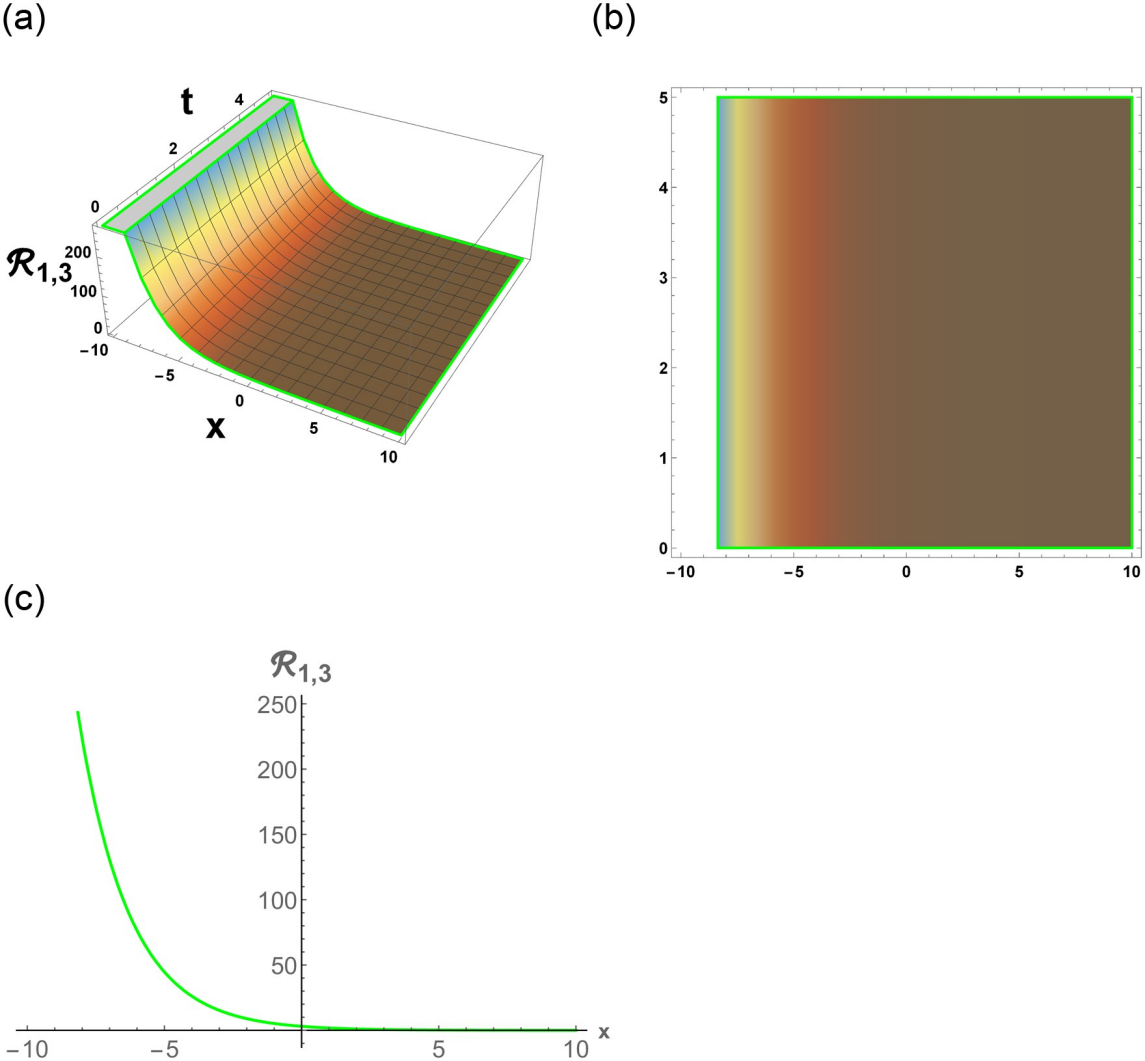
Physical depiction of $R_{1,3}$ under parametric values are $h_{1}=1.2,\;h_{2}=1.02,\;\gamma =0.03,\;\theta =0.01,\;\beta =0.006,\;U=1.02,\;p=1.01\;\text{and}\;\tau =1.3$. (a) 3-D, (b) Density, (c) 2-D.

**Fig 5:**
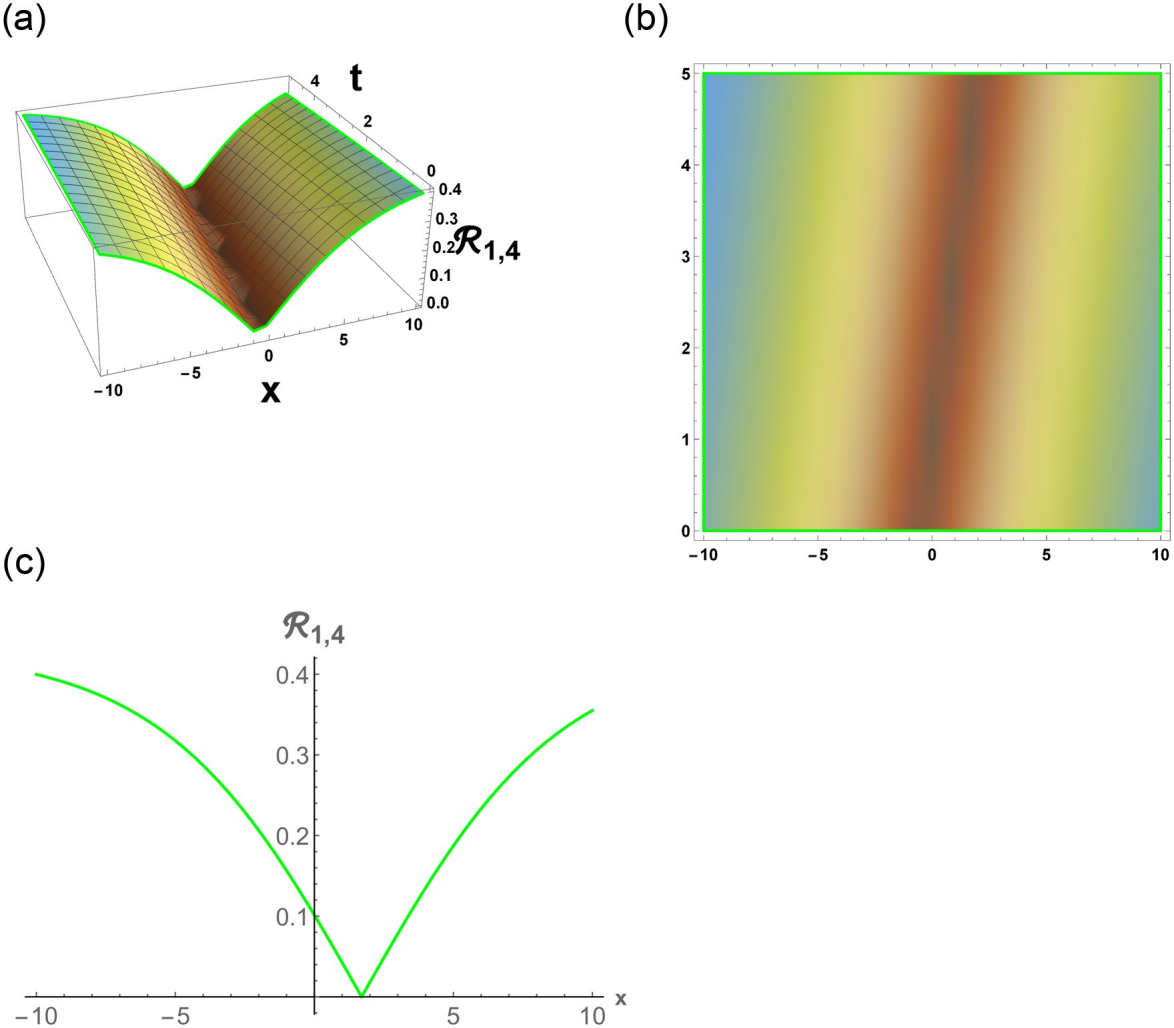
Physical depiction of $R_{1,4}$ under parametric values are $h_{1}=0.12,\;h_{2}=1.12,\;\gamma =0.13,\;\theta =0.11,\;\beta =0.16,\;U=1.12,\;p=1.11\;\text{and}\;\tau =0.13$. (a) 3-D, (b) Density, (c) 2-D.

**Fig 6:**
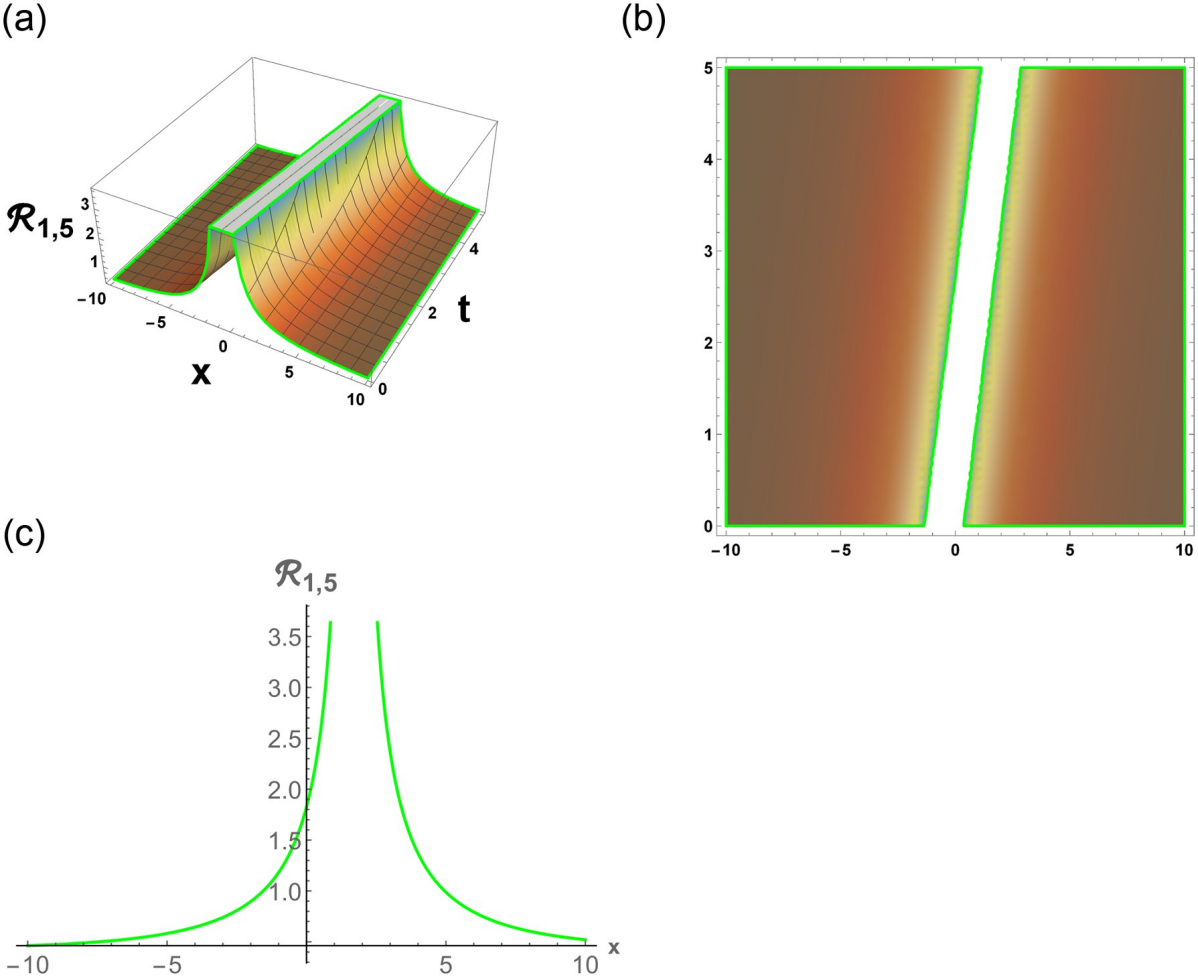
Physical depiction of $R_{1,5}$ under parametric values are $h_{1}=0.22,\;h_{2}=1.22,\;\gamma =0.23,\;\theta =0.21,\;\beta =0.26,\;U=1.22,\;p=1.21\;\text{and}\;\tau =0.23$. (a) 3-D, (b) Density, (c) 2-D.

Fig 1, represent the flow chart of the employed method. Fig 2, represents the bright stochastic soliton. Fig 3, represents the singular stochastic soliton. Fig 4, represents the hyperbolic stochastic soliton. Fig 5, represents the dark stochastic soliton. Fig 6, represents the bell-shaped singular stochastic soliton.

**Fig 7:**
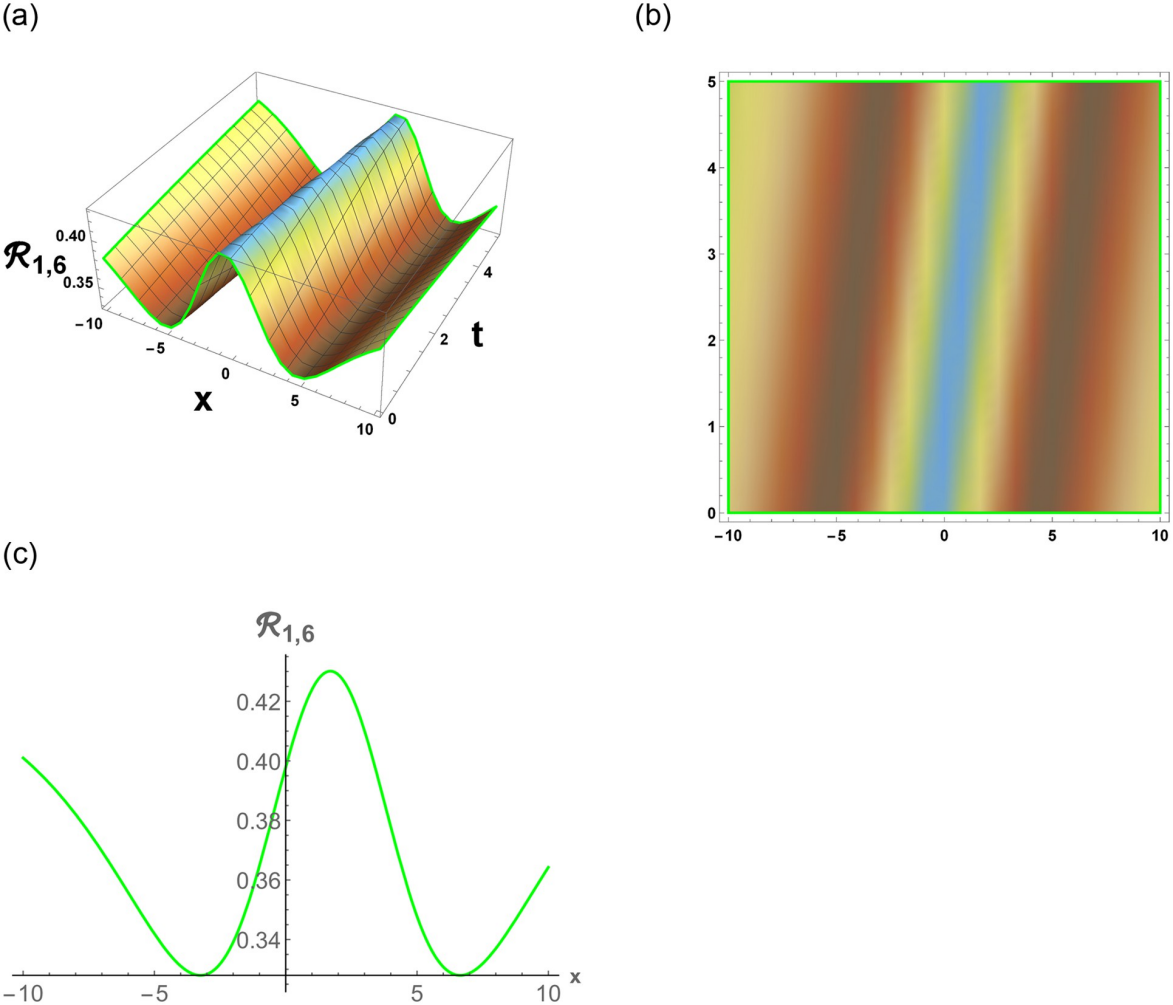
Physical depiction of $R_{1,6}$ under parametric values are $h_{1}=0.42,\;h_{2}=1.42,\;\gamma =0.43,\;\theta =0.41,\;\beta =0.46,\;U=1.42,\;p=1.41\;\text{and}\;\tau =0.43$. (a) 3-D, (b) Density, (c) 2-D.

**Fig 8:**
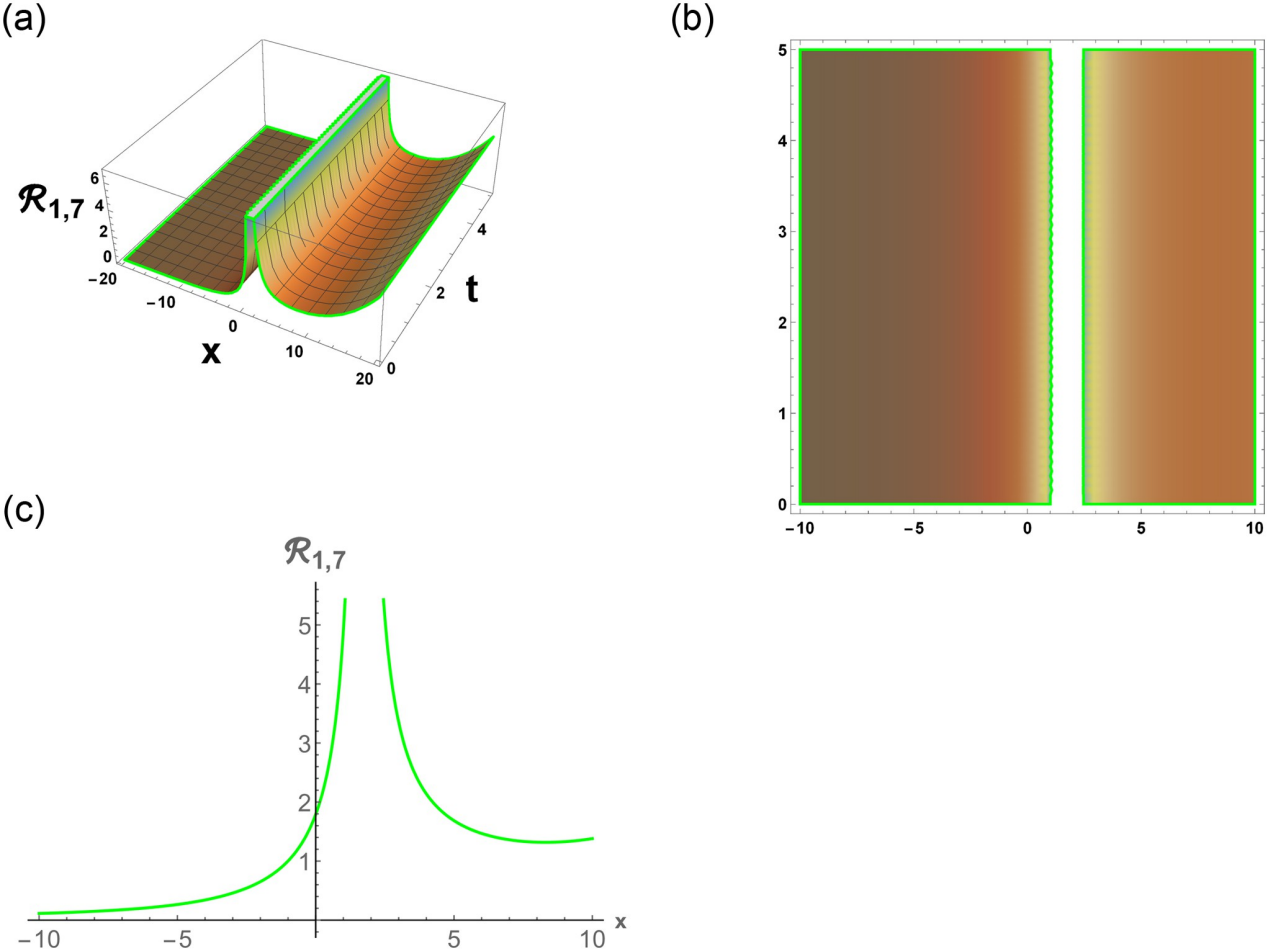
Physical depiction of $R_{1,7}$ under parametric values are $h_{1}=0.52,\;h_{2}=1.52,\;\gamma =0.53,\;\theta =0.51,\;\beta =0.56,\;U=1.52,\;p=1.51\;\text{and}\;\tau =0.53$. (a) 3-D, (b) Density, (c) 2-D.

**Fig 9:**
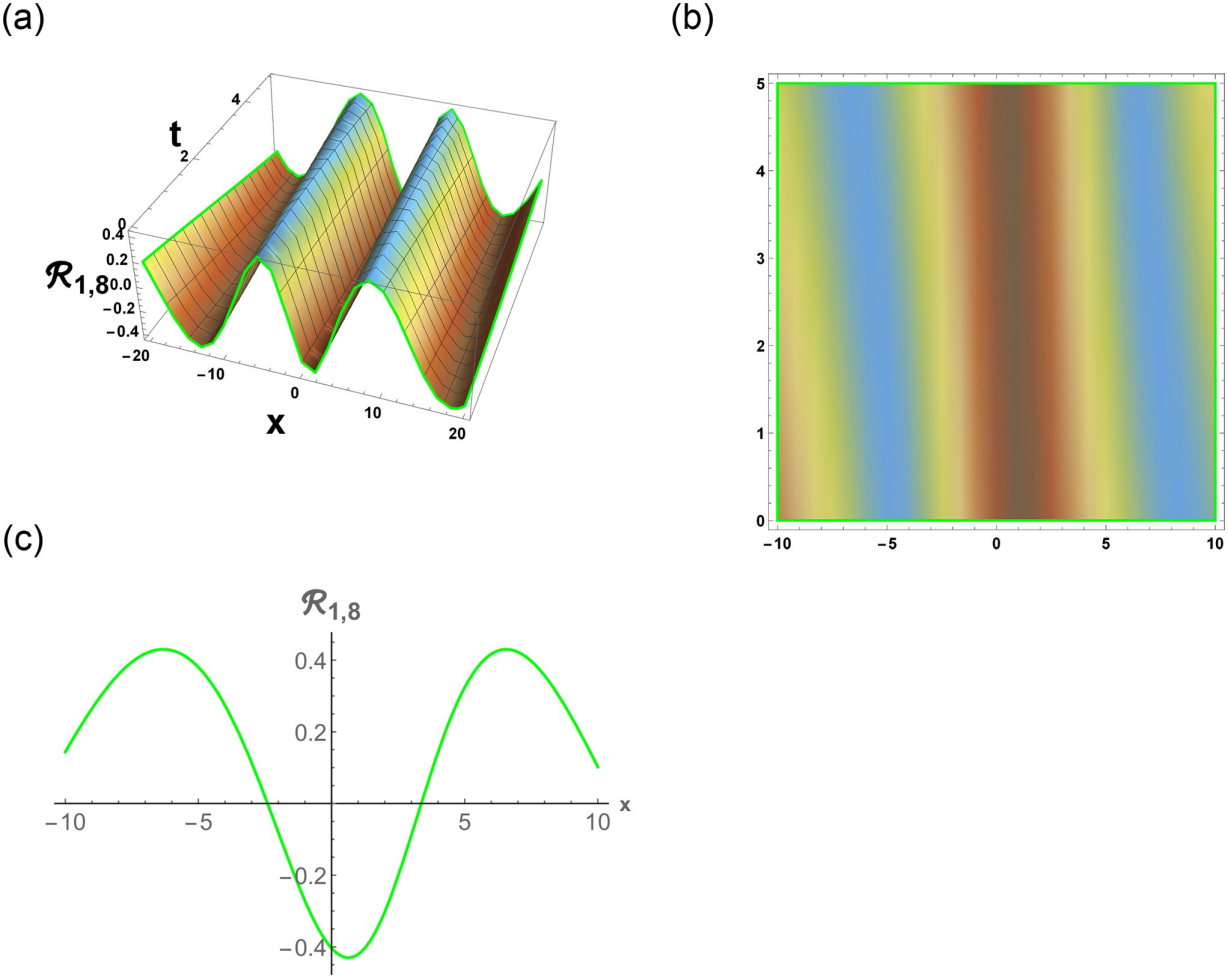
Physical depiction of $R_{1,8}$ under parametric values are $h_{1}=0.62,\;h_{2}=1.62,\;\gamma =0.63,\;\theta =0.61,\;\beta =0.66,\;U=1.62,\;p=1.61\;\text{and}\;\tau =0.63$. (a) 3-D, (b) Density, (c) 2-D.

**Fig 10:**
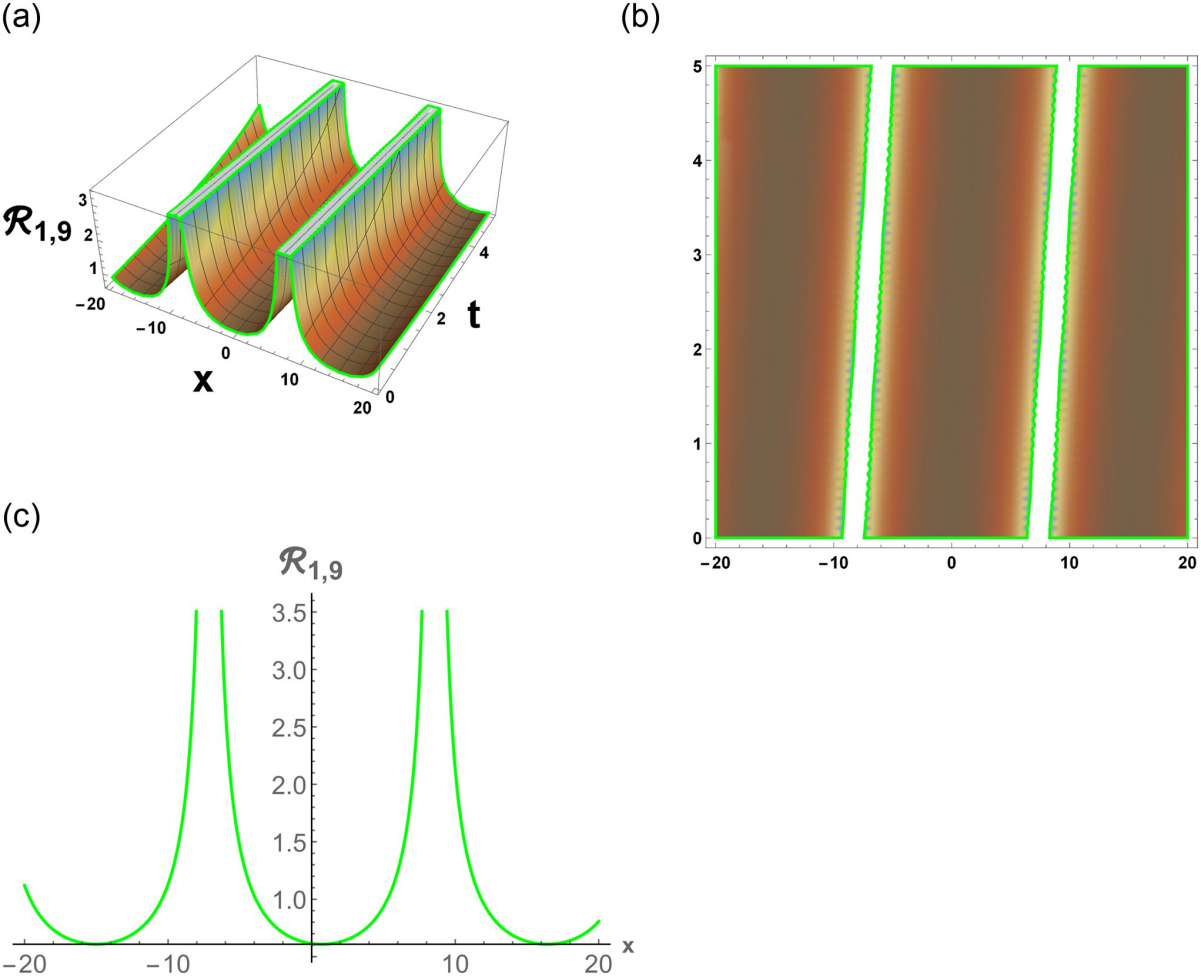
Physical depiction of $R_{1,9}$ under parametric values are $h_{1}=0.82,\;h_{2}=1.82,\;\gamma =0.83,\;\theta =0.81,\;\beta =0.86,\;U=1.82,\;p=1.81\;\text{and}\;\tau =0.83$. (a) 3-D, (b) Density, (c) 2-D.

**Fig 11:**
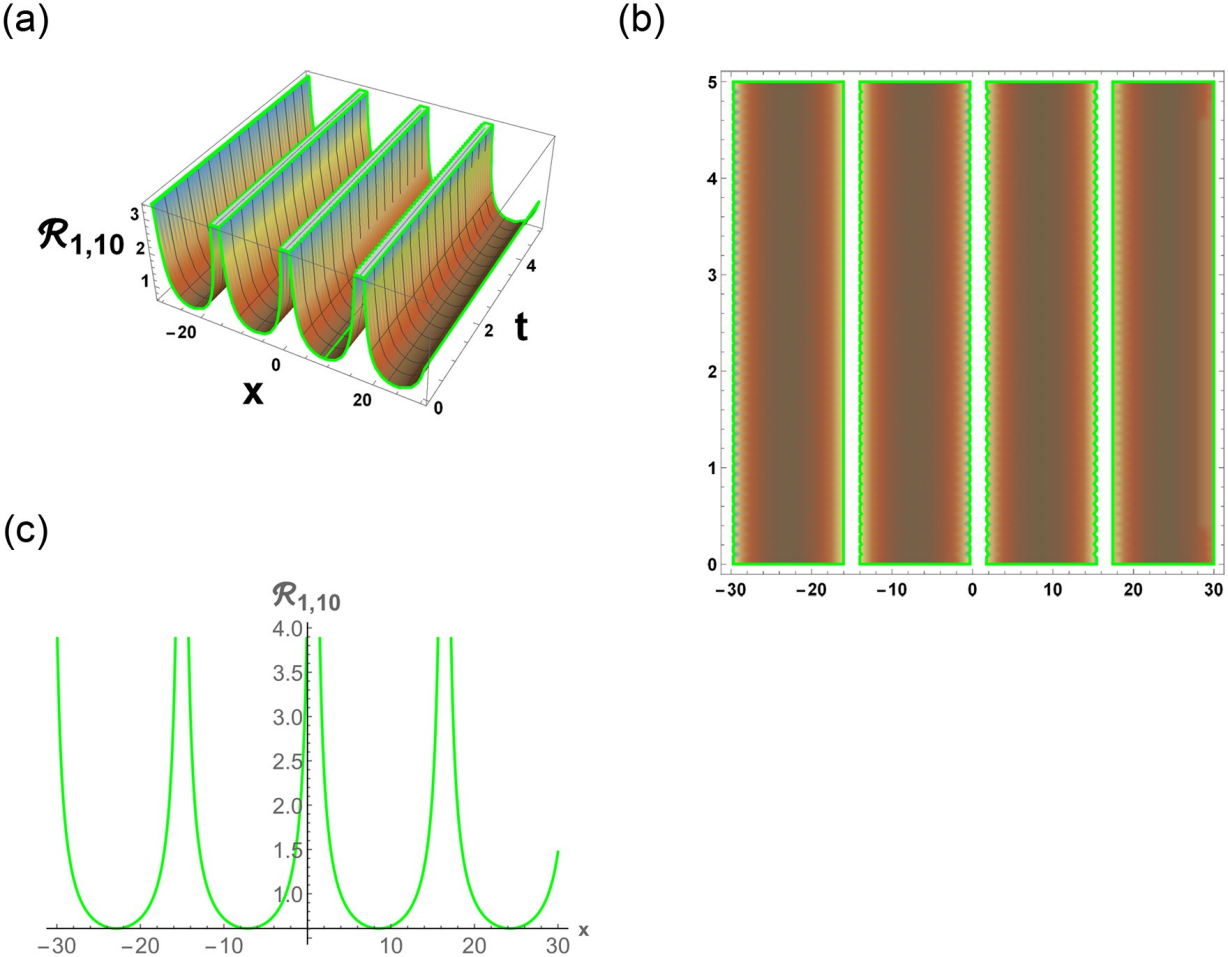
Physical depiction of $R_{1,100}$ under parametric values are $h_{1}=0,\;h_{2}=1.2,\;\gamma =0.3,\;\theta =0.1,\;\beta =0.6,\;U=1.2,\;p=1.1\;\text{and}\;\tau =0.3$. (a) 3-D, (b) Density, (c) 2-D.

Fig 7, represents the combo of bright and dark stochastic soliton. Fig 8, represents the singular and dark stochastic soliton. Fig 9, represents the periodic stochastic soliton. Figs 10 and 11, represents the trigonometric stochastic soliton.

Figs 12 and 13, represents the exponential stochastic soliton. Figs 14 and 15, represents the plane wave stochastic soliton. Figs 16–19, represents the phase portrait analysis without perturbation term. Fig 20, represents the phase portrait analysis with perturbation term.

**Fig 12:**
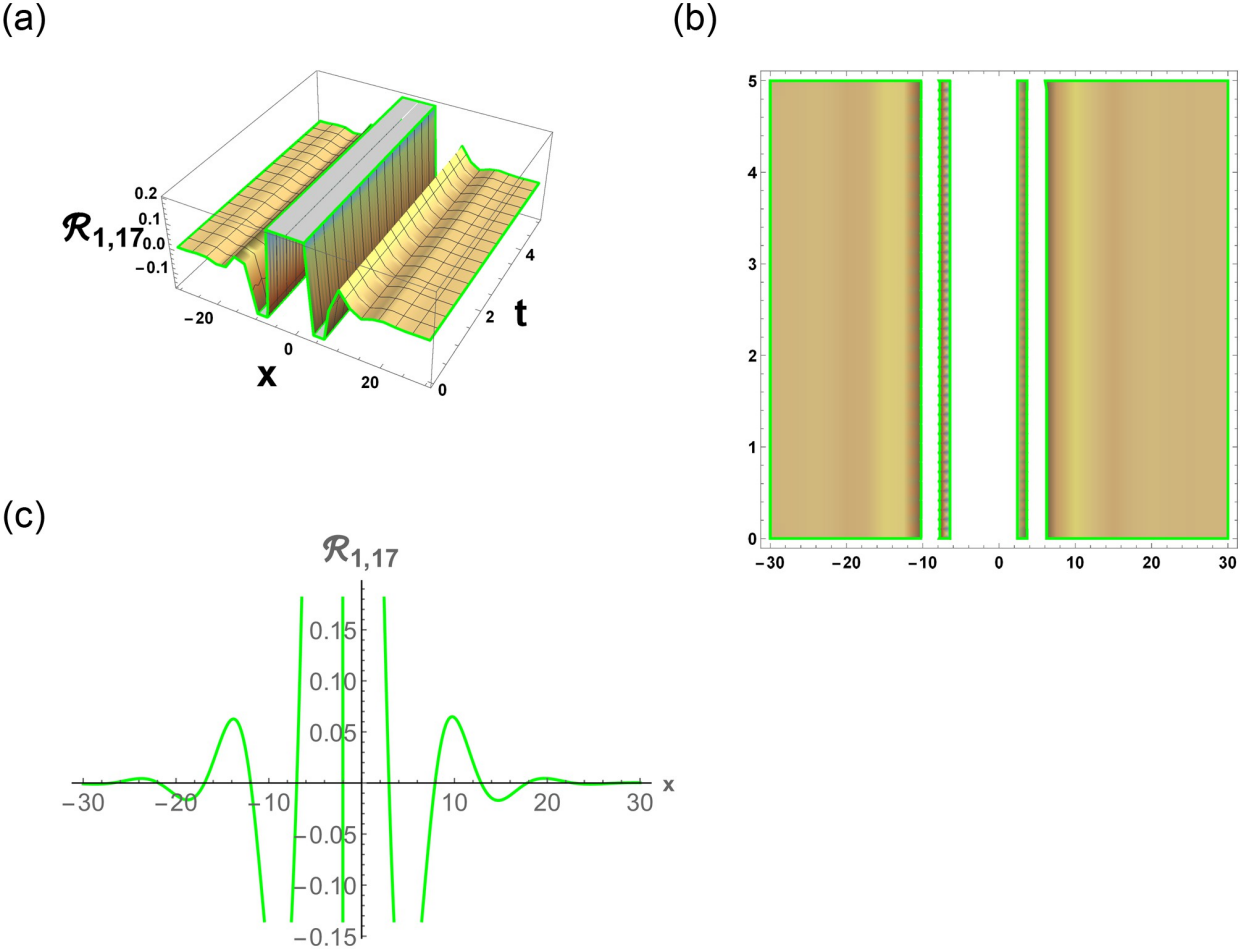
Physical depiction of $R_{1,17}$ under parametric values are $h_{1}=0,\;h_{2}=1.02,\;\gamma =1.3,\;\theta =1.1,\;\beta =1.6,\;U=0.2,\;p=0.1\;\text{and}\;\tau =1.3$. (a) 3-D, (b) Density, (c) 2-D.

**Fig 13:**
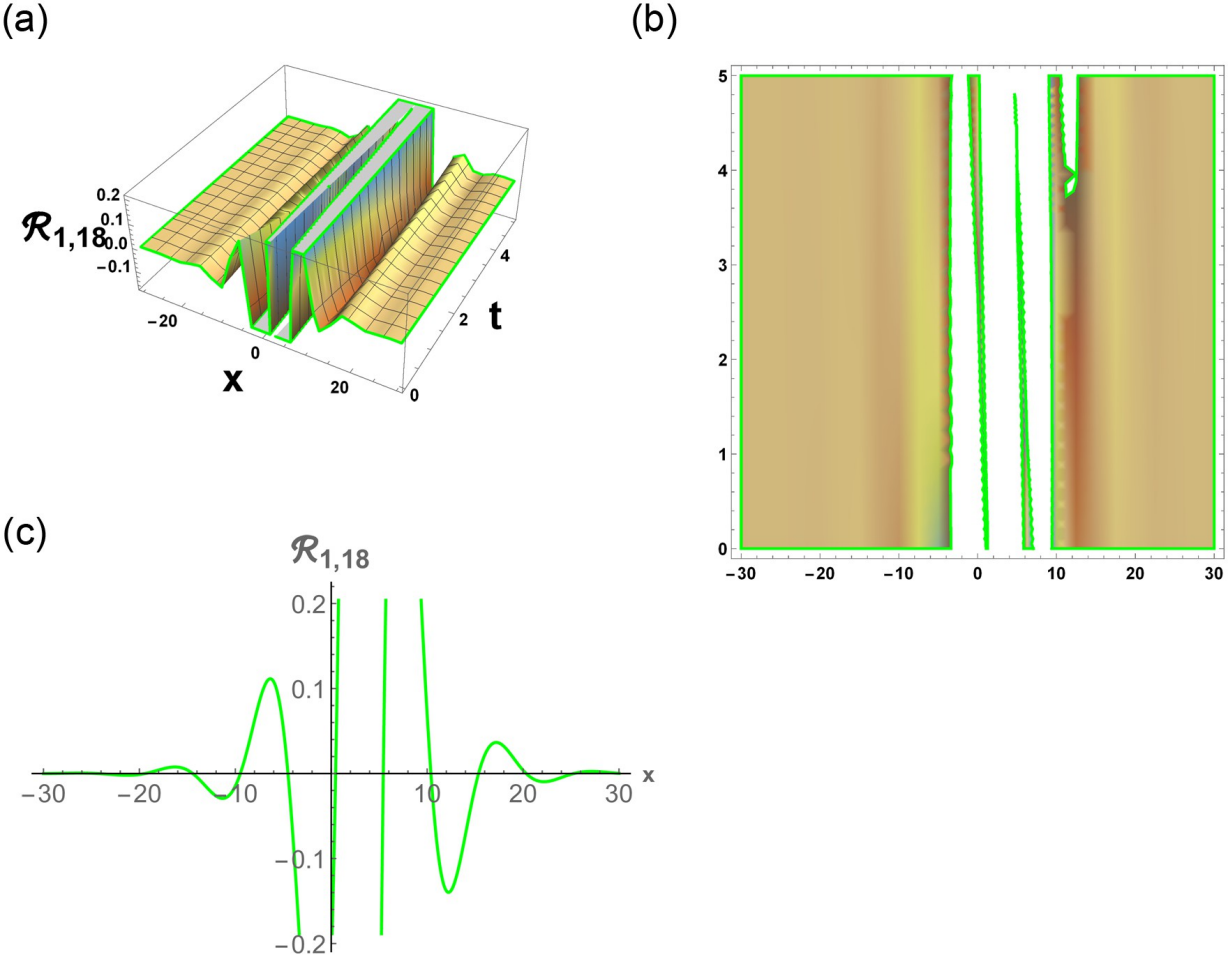
Physical depiction of $R_{1,18}$ under parametric values are $h_{1}=0,\;h_{2}=1.12,\;\gamma =0.13,\;\theta =0.11,\;\beta =0.16,\;U=1.12,\;p=1.11\;\text{and}\;\tau =0.13$. (a) 3-D, (b) Density, (c) 2-D.

**Fig 14:**
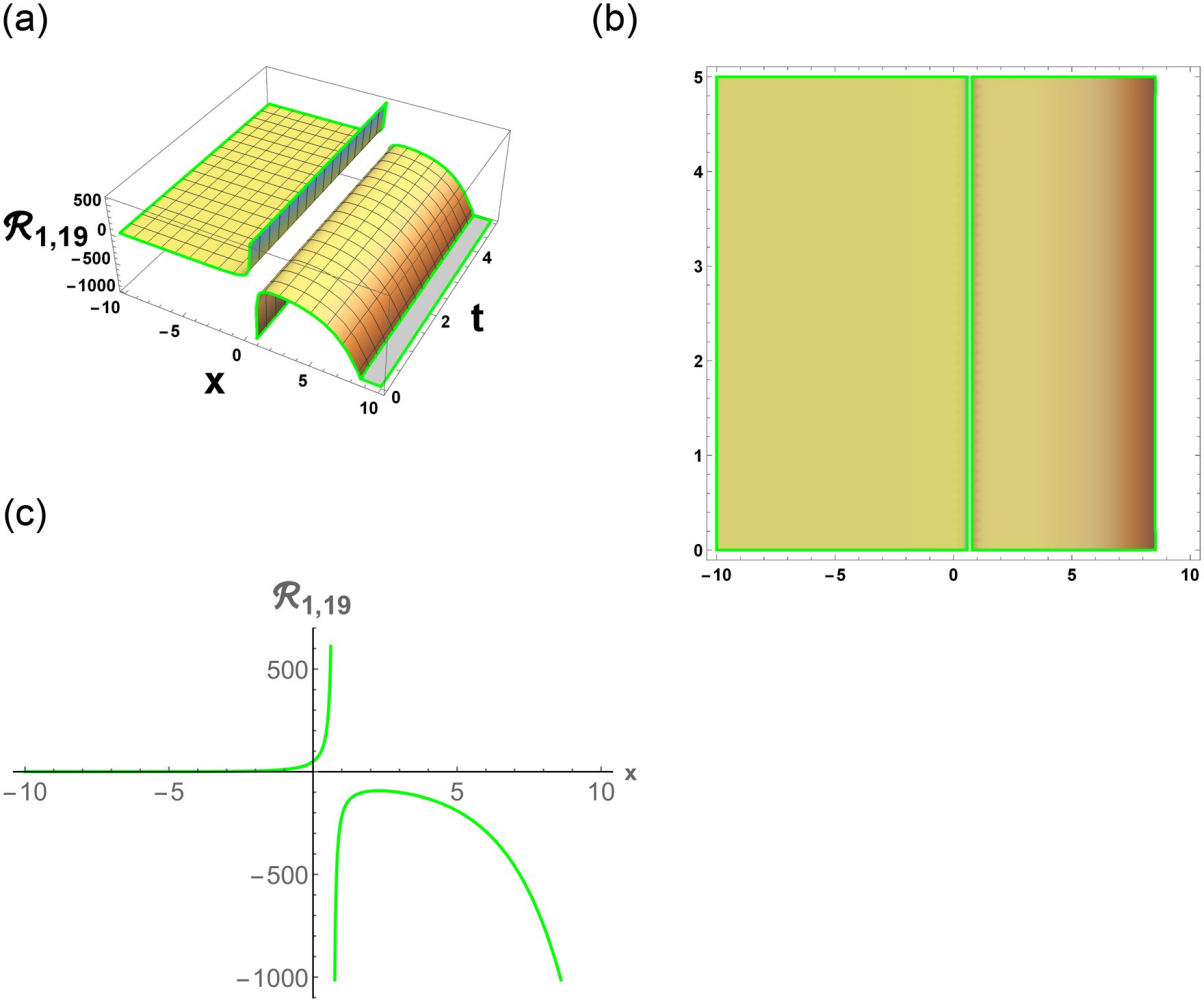
Physical depiction of $R_{1,19}$ under parametric values are $h_{1}=1.02,\;h_{2}=1.02,\;\gamma =0.03,\;\theta =0.01,\;\beta =0.06,\;U=1.02,\;p=1.01\;\text{and}\;\tau =0.05$. (a) 3-D, (b) Density, (c) 2-D.

**Fig 15:**
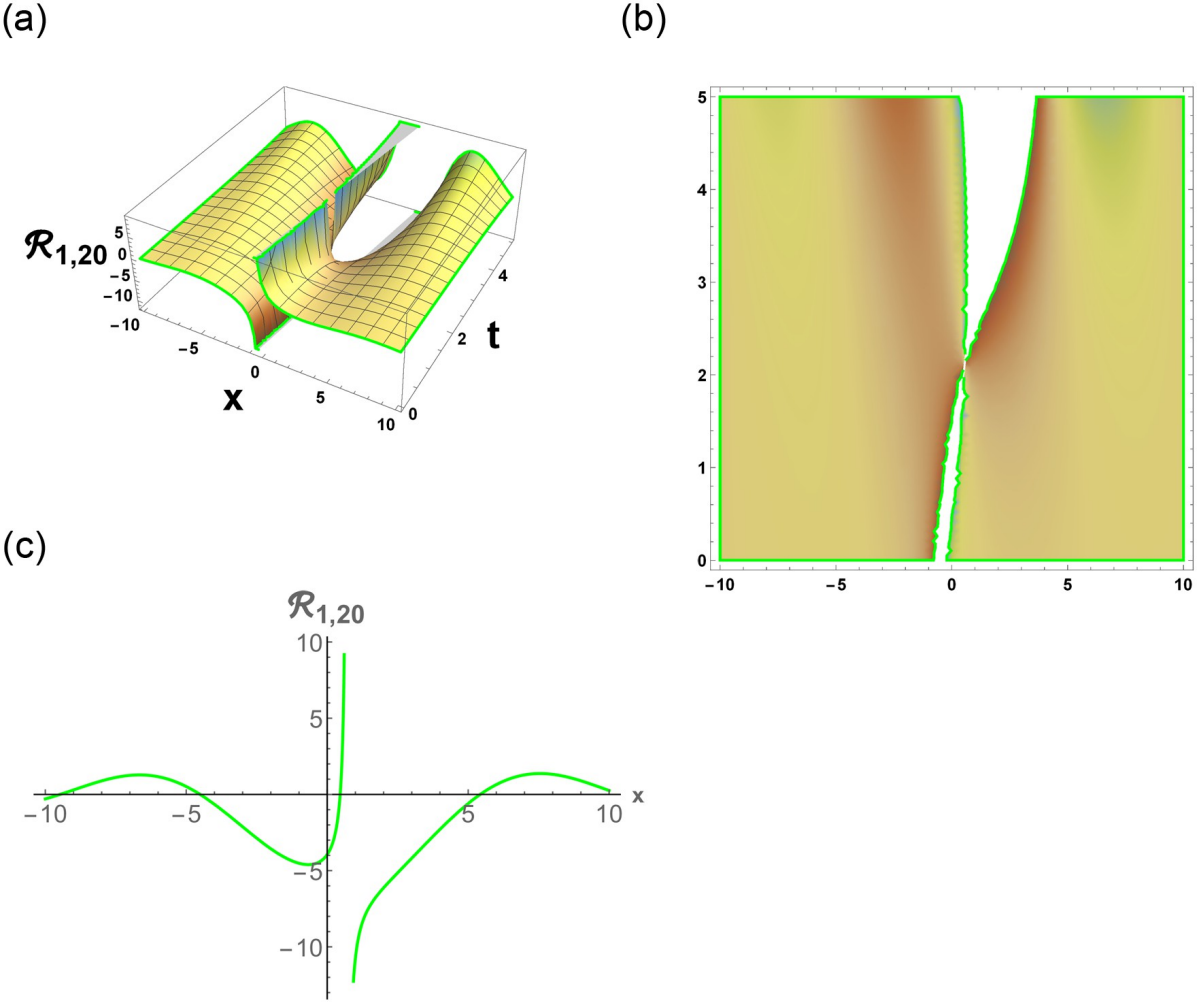
Physical depiction of $R_{1,20}$ under parametric values are $h_{1}=1,\;h_{2}=0.2,\;\gamma =0.3,\;\theta =0.1,\;\beta =0.6,\;U=1.2,\;p=1.1\;\text{and}\;\tau =0.3$. (a) 3-D, (b) Density, (c) 2-D.

**Fig 16:**
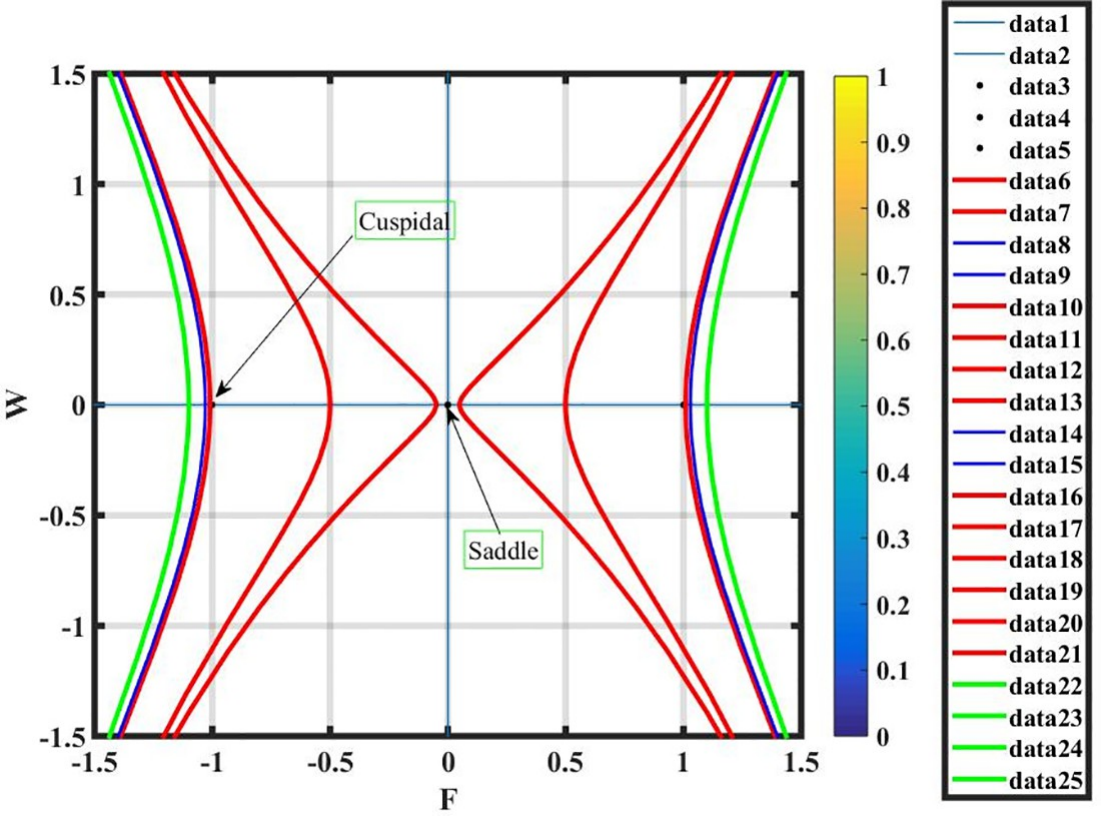
Phase plane analysis without perturbation term of case-i, at suitable parametric conditions.

**Fig 17:**
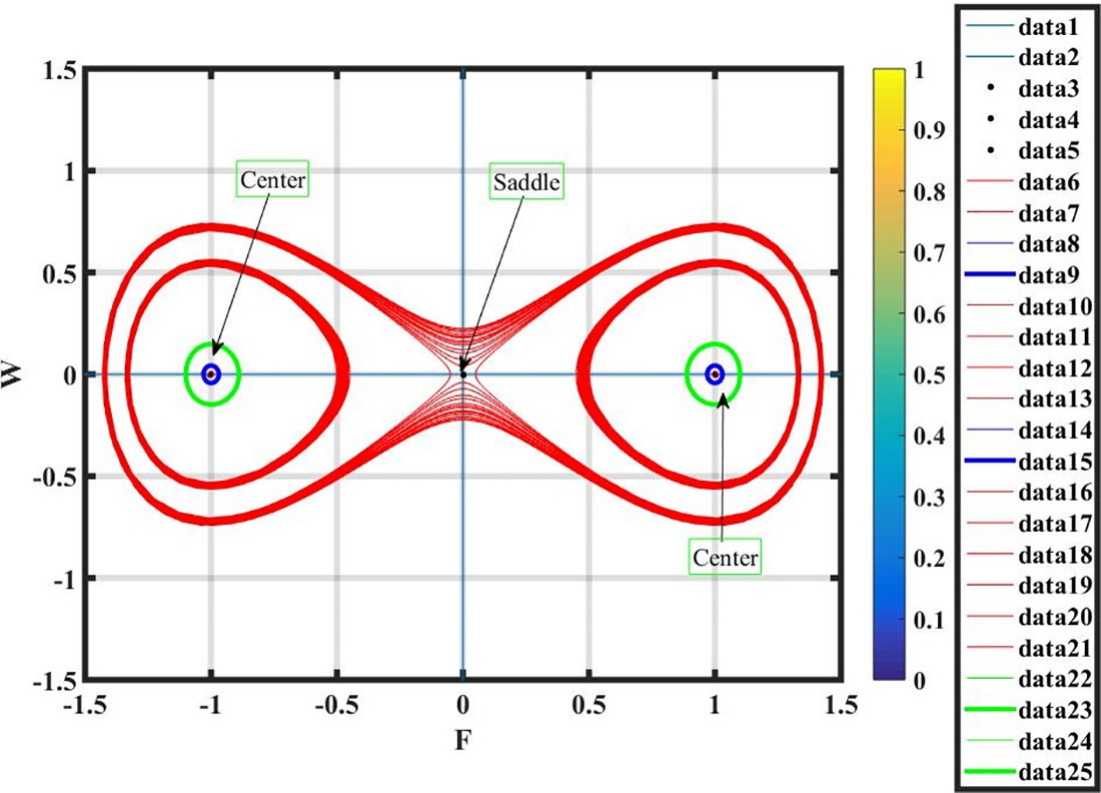
Phase plane analysis without perturbation term of case-ii, at suitable parametric conditions.

**Fig 18:**
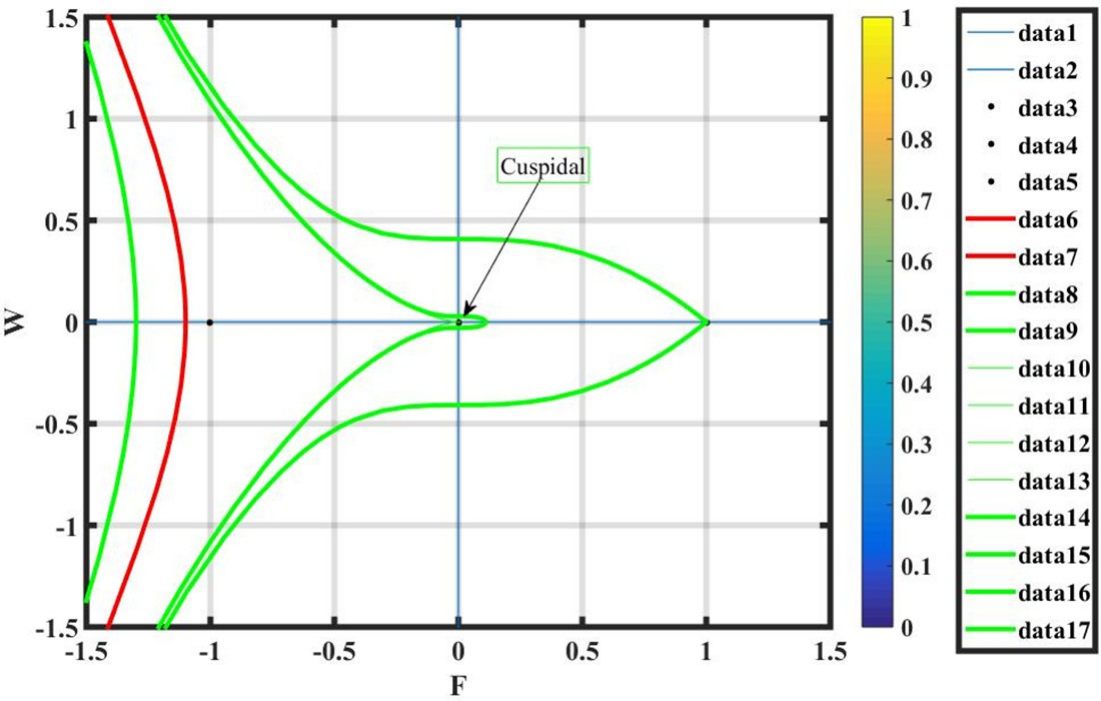
Phase plane analysis without perturbation term of case-iii, at suitable parametric conditions.

**Fig 19:**
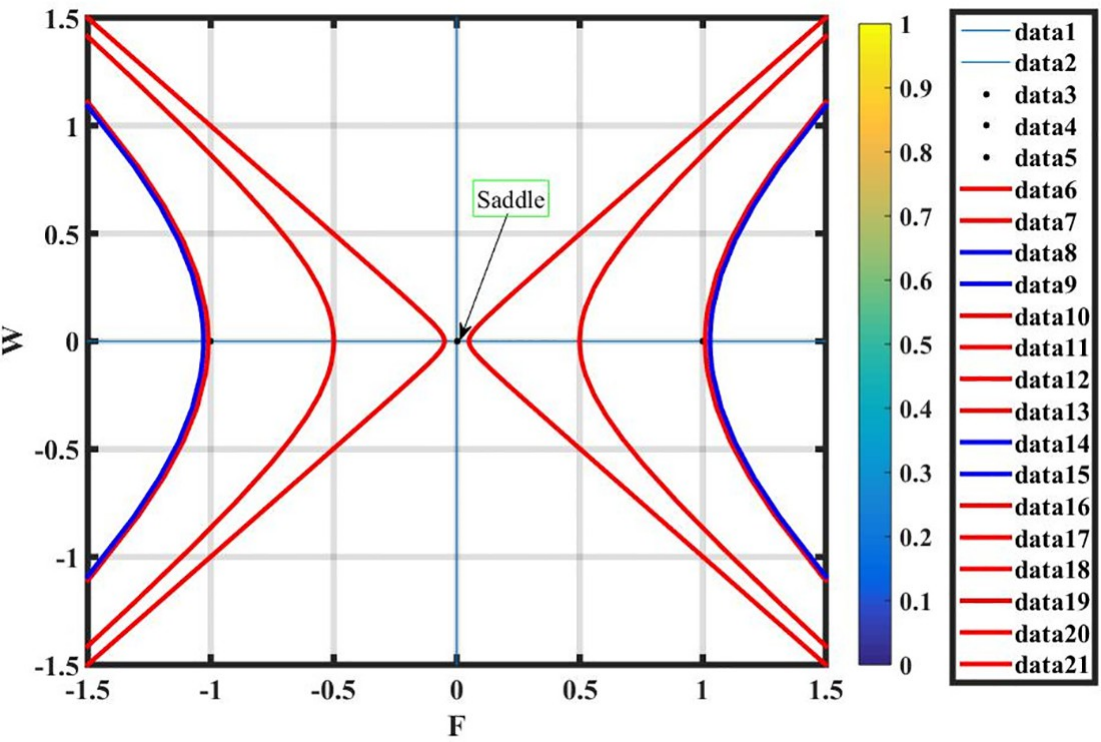
Phase plane analysis without perturbation term of case-iv, at suitable parametric conditions.

**Fig 20:**
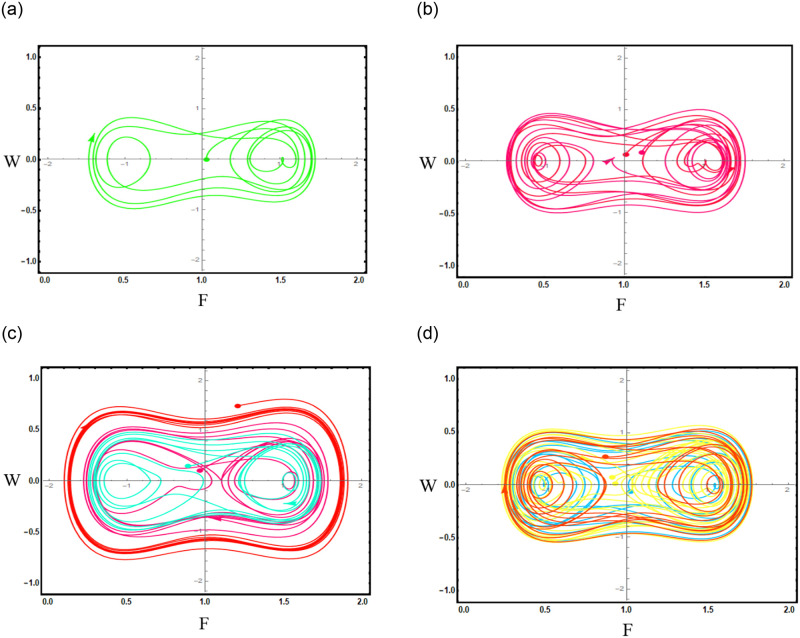
Phase plane analysis with perturbation term of Eq (64), at suitable parametric conditions. (a) Phase plane analysis with perturbation term when *ξ* = 0.3, (b) Phase plane analysis with perturbation term when *ξ* = 1, (c) Phase plane analysis with perturbation term when *ξ* = 1.5, (d) Phase plane analysis with perturbation term when *ξ* = 2.3.

**Fig 21:**
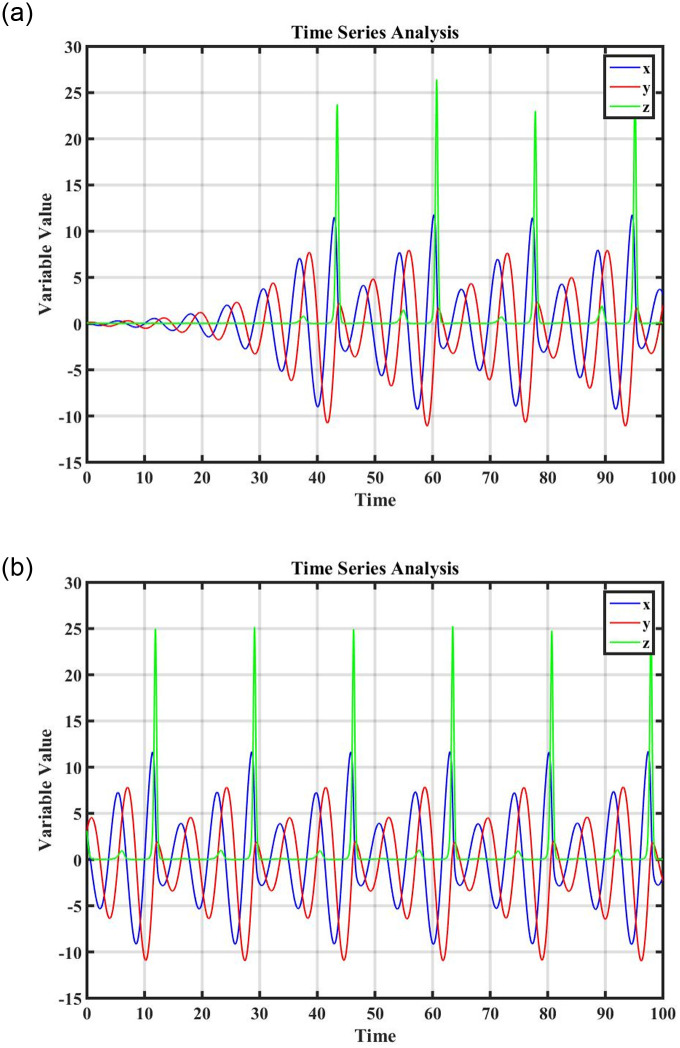
Time series analysis of dynamical system (61), at (0, 0.4), (0, 0.7) and (0, 0.9), initial conditions. (a) Time series analysis when *ξ* = 1.3, (b) Time series analysis when *ξ* = 1.6.

**Fig 22:**
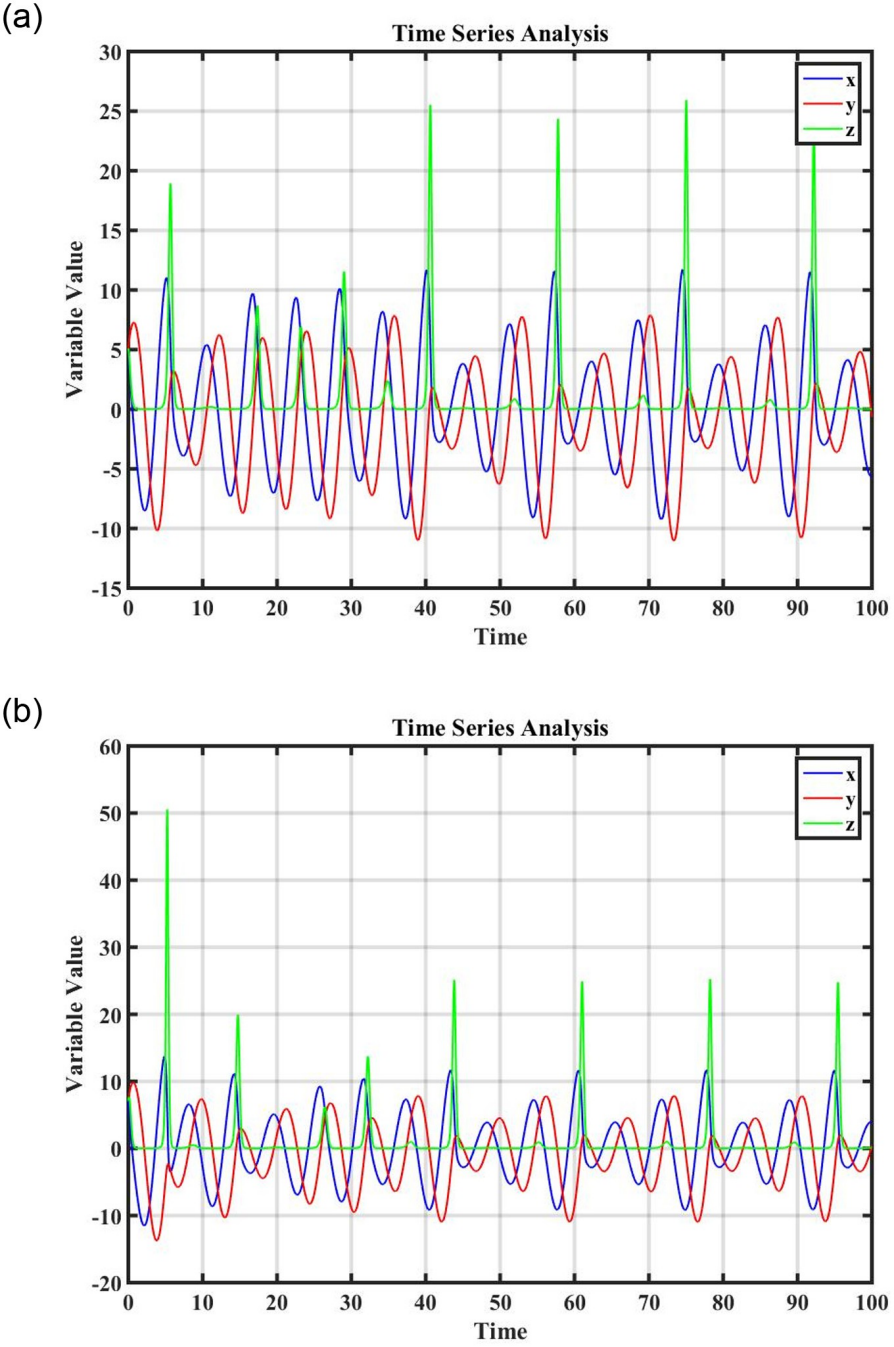
Time series analysis of dynamical system (64), at (0, 1.4), (0, 4.7) and (0, 7.9), initial conditions. (a) Time series analysis when *ξ* = 2, (b) Time series analysis when *ξ* = 2.3.

**Fig 23:**
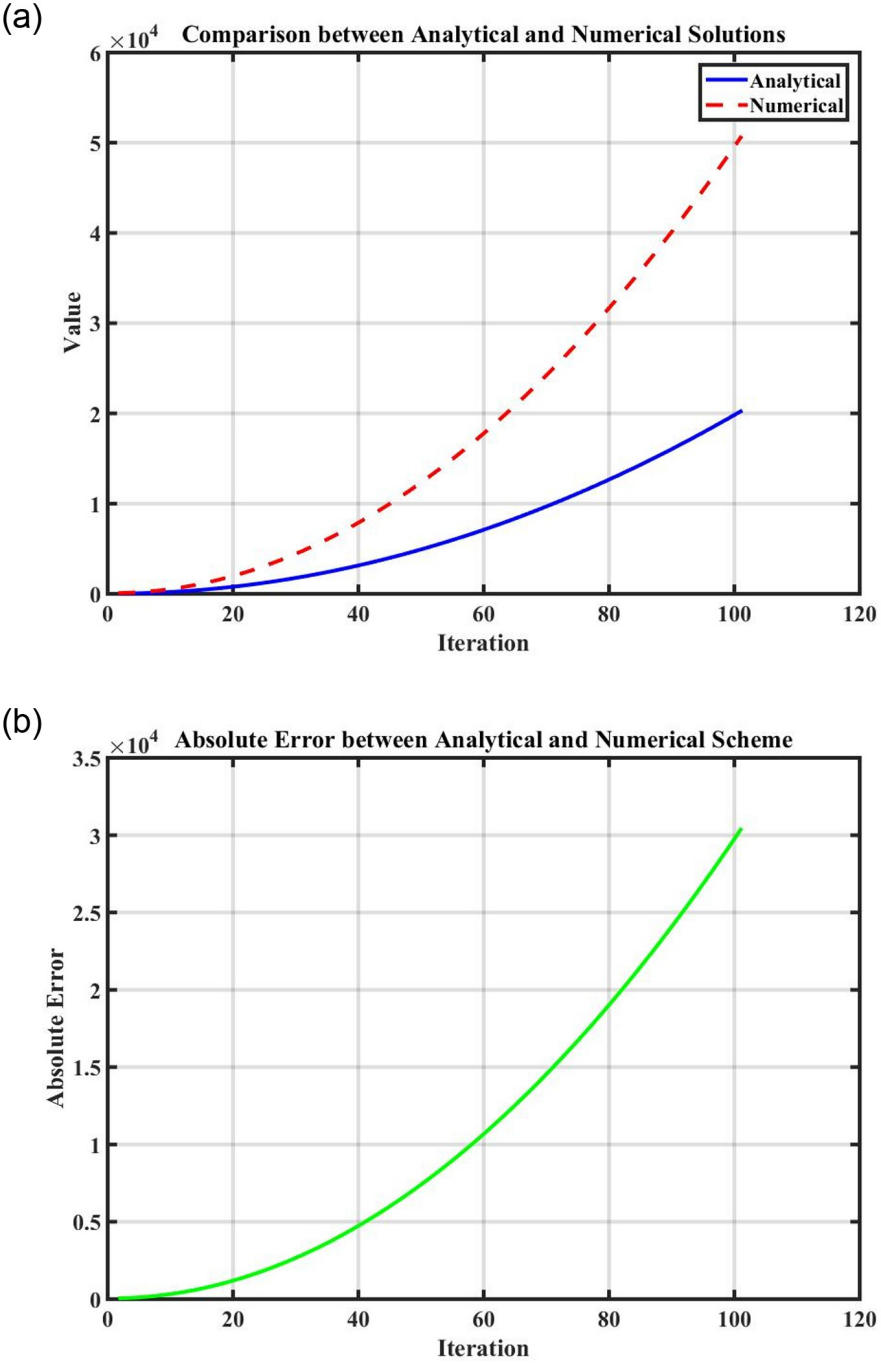
Comparison of numerical simulation and analytical exact solutions with error term using the numerical values of Table 1. (a) Comparison of analytical and numerical solution, (b) Error term.

**Fig 24:**
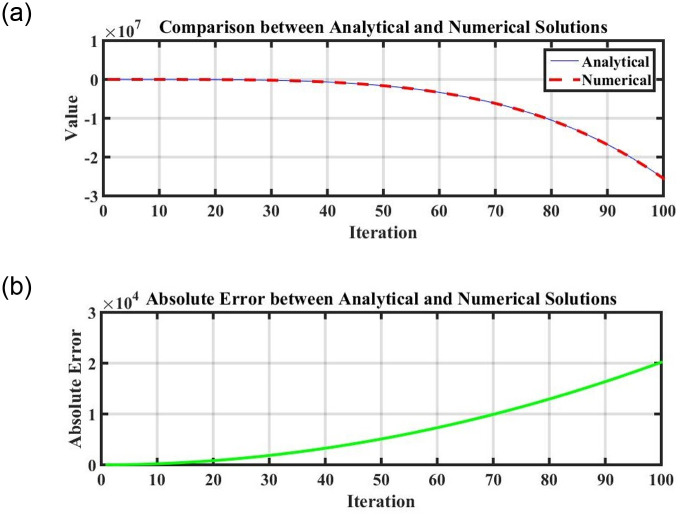
Comparison of numerical simulation and analytical exact solutions with error term using the numerical values of Tables 2 and 3. (a) Comparison of analytical and numerical solution, (b) Error term.

**Fig 25:**
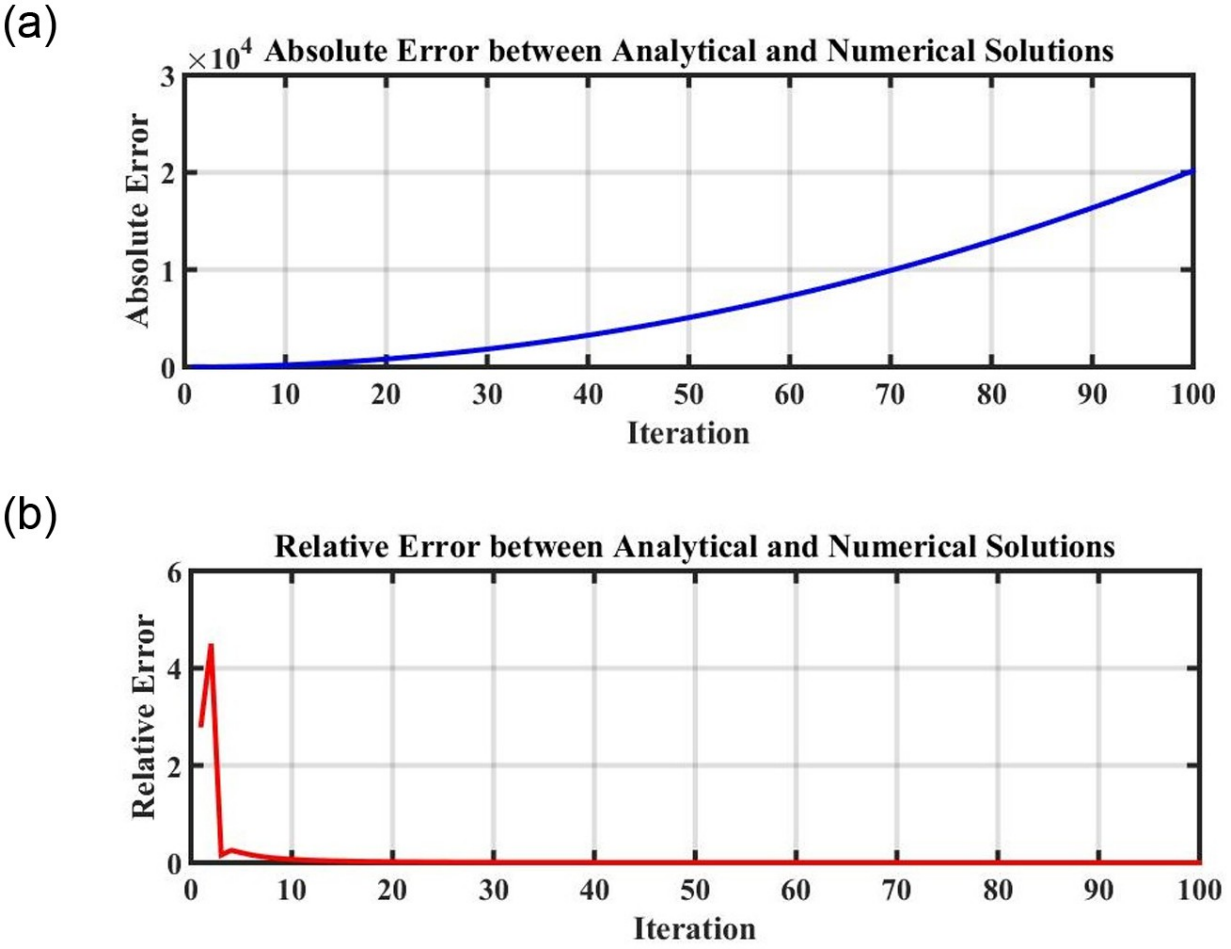
Absolute and relative error of the obtained error between analytical and numerical stochastic solutions. (a) Absolute error, (b) Relative error.

Figs 21 and 22, represents the time series analysis of the dynamical system. Figs 23 and 24, represents the comparison of analytical and numerical solutions and error terms. Fig 25, represents the relative and absolute error terms.

**Table 1:** Comparison of analytical solutions via the MSSE technique and numerical solutions computed via the modified VI technique for the model under investigation.

Iteration	Analytical	Numerical	Absolute Error	Relative Error
1	6	23	17	2.8333
2	2	11	9	4.5
3	-19	-22	3	0.15789
4	-75	-94	19	0.25333
5	-190	-229	39	0.20526
6	-394	-457	63	0.1599
7	-723	-814	91	0.12586
8	-1219	-1342	123	0.1009
9	-1930	-2089	159	0.082383
10	-2910	-3109	199	0.068385
11	-4219	-4462	243	0.057597
12	-5923	-6214	291	0.049131
13	-8094	-8437	343	0.042377
14	-10810	-11209	399	0.03691
15	-14155	-14614	459	0.032427
16	-18219	-18742	523	0.028706
17	-23098	-23689	591	0.025587
18	-28894	-29557	663	0.022946
19	-35715	-36454	739	0.020692
20	-43675	-44494	819	0.018752
21	-52894	-53797	903	0.017072
22	-63498	-64489	991	0.015607
23	-75619	-76702	1083	0.014322
24	-89395	-90574	1179	0.013189
25	−1.0497*e*+ 05	−1.0625*e*+ 05	1279	0.012184
26	−1.2249*e* + 05	−1.2388*e* + 05	1383	0.01129
27	−1.4212*e* + 05	−1.4361*e* + 05	1491	0.010491
28	−1.6402*e* + 05	−1.6562*e* + 05	1603	0.0097733
29	−1.8835*e* + 05	−1.9007*e* + 05	1719	0.0091266
30	−2.1529*e* + 05	−2.1713*e* + 05	1839	0.008542
31	−2.4502*e* + 05	−2.4698*e* + 05	1963	0.0080116
32	−2.7772*e* + 05	−2.7981*e* + 05	2091	0.0075291
33	−3.1359*e* + 05	−3.1582*e* + 05	2223	0.0070888
34	−3.5283*e* + 05	−3.5519*e* + 05	2359	0.0066859
35	−3.9564*e* + 05	−3.9813*e* + 05	2499	0.0063164
36	−4.4222*e* + 05	−4.4486*e* + 05	2643	0.0059767
37	−4.928*e* + 05	−4.9559*e* + 05	2791	0.0056636
38	−5.4759*e* + 05	−5.5054*e* + 05	2943	0.0053744
39	−6.0684*e* + 05	−6.0993*e* + 05	3099	0.0051068
40	−6.7076*e* + 05	−6.7401*e* + 05	3259	0.0048587

**Table 2:** Comparison of techniques for the model under investigation.

Iteration	Analytical	Numerical	Absolute Error	Relative Error
41	−7.3959*e* + 05	−7.4302*e* + 05	3423	0.0046282
42	−8.136*e* + 05	−8.1719*e* + 05	3591	0.0044137
43	−8.9302*e* + 05	−8.9678*e* + 05	3763	0.0042138
44	−9.7812*e* + 05	−9.8205*e* + 05	3939	0.0040271
45	−1.0692e + 06	−1.0733*e* + 06	4119	0.0038526
46	−1.1664*e* + 06	−1.1707*e* + 06	4303	0.0036891
47	−1.2701*e* + 06	−1.2746*e* + 06	4491	0.0035359
48	−1.3806*e* + 06	−1.3853*e* + 06	4683	0.003392
49	−1.4982e + 06	−1.503*e* + 06	4879	0.0032566
50	−1.6231*e* + 06	−1.6281e + 06	5079	0.0031293
51	−1.7556e + 06	−1.7609*e* + 06	5283	0.0030092
52	−1.8961*e* + 06	−1.9016*e* + 06	5491	0.0028959
53	−2.0449*e* + 06	−2.0506*e* + 06	5703	0.0027889
54	−2.2023*e* + 06	−2.2082*e* + 06	5919	0.0026877
55	−2.3685*e* + 06	−2.3747*e* + 06	6139	0.0025919
56	−2.544e + 06	−2.5504*e* + 06	6363	0.0025012
57	−2.7291*e* + 06	−2.7357*e* + 06	6591	0.0024151
58	−2.9241*e* + 06	−2.9309*e* + 06	6823	0.0023334
59	−3.1294*e* + 06	−3.1364*e* + 06	7059	0.0022557
60	−3.3452*e* + 06	−3.3525*e* + 06	7299	0.0021819
61	−3.5721*e* + 06	−3.5796*e* + 06	7543	0.0021116
62	−3.8103*e* + 06	−3.8181*e* + 06	7791	0.0020447
63	−4.0602*e* + 06	−4.0683*e* + 06	8043	0.0019809
64	−4.3222*e* + 06	−4.3305*e* + 06	8299	0.0019201
65	−4.5967*e* + 06	−4.6053*e* + 06	8559	0.001862
66	−4.8841*e* + 06	−4.8929*e* + 06	8823	0.0018065
67	−5.1847*e* + 06	−5.1938*e* + 06	9091	0.0017534
68	−5.499*e* + 06	−5.5084*e* + 06	9363	0.0017027
69	−5.8274*e* + 06	−5.837*e* + 06	9639	0.0016541
70	−6.1703*e* + 06	−6.1802*e* + 06	9919	0.0016076
71	−6.528e + 06	−6.5382*e* + 06	10203	0.001563
72	−6.9011*e* + 06	−6.9116*e* + 06	10491	0.0015202
73	−7.29e + 06	−7.3008e + 06	10783	0.0014792
74	−7.6951e + 06	−7.7061*e* + 06	11079	0.0014398
75	−8.1168*e* + 06	−8.1282*e* + 06	11379	0.0014019
76	−8.5556*e* + 06	−8.5673*e* + 06	11683	0.0013655
77	−9.012*e* + 06	−9.024*e* + 06	11991	0.0013306
78	−9.4864*e* + 06	−9.4987*e* + 06	12303	0.0012969
79	−9.9793*e* + 06	−9.9919*e* + 06	12619	0.0012645
80	−1.0491*e* + 07	−1.0504*e* + 07	12939	0.0012333

**Table 3:** Comparison of techniques for the model under investigation.

Iteration	Analytical	Numerical	Absolute Error	Relative Error
81	−1.1022e + 07	−1.1036e + 07	13263	0.0012033
82	−1.1574e + 07	−1.1587e + 07	13591	0.0011743
83	−1.2145e + 07	−1.2159e + 07	13923	0.0011464
84	−1.2738e + 07	−1.275e + 07	14259	0.0011194
85	−1.3352e + 07	−1.3366e + 07	14599	0.0010934
86	−1.3988e + 07	−1.4003e + 07	14943	0.0010683
87	−1.4646e + 07	−1.4661e + 07	15291	0.001044
88	−1.5327e + 07	−1.5343e + 07	15643	0.0010206
89	−1.6032e + 07	−1.6048e + 07	15999	0.00099794
90	−1.6761e + 07	−1.6777e + 07	16359	0.00097603
91	−1.7514e + 07	−1.7531e + 07	16723	0.00095482
92	−1.8293e + 07	−1.831e + 07	17091	0.00093431
93	−1.9097e + 07	−1.9114e + 07	17463	0.00091444
94	−1.9927e + 07	−1.9945e + 07	17839	0.0008952
95	−2.0784e + 07	−2.0803e + 07	18219	0.00087657
96	−2.1669e + 07	−2.1688e + 07	18603	0.00085851
97	−2.2581e + 07	−2.26e + 07	18991	0.000841
98	−2.3522e + 07	−2.3542e + 07	19383	0.00082402
99	−2.4493e + 07	−2.4512e + 07	19779	0.00080755
100	−2.5492e + 07	−2.5513e + 07	20179	0.00079157

### Physical description

The figures are plotted against 3-D, 2-D and density graphs at suitable free parameters in the solutions. The SNLSM is then transformed into a system of planar dynamics using the stochastic wave method. The behavior of the considered equation in dynamic and chaotic conditions has been thoroughly discussed. Utilizing the ideas of bifurcation and techniques for phase portrait interpretation, the bifurcations for a planar system of dynamics Eq (61) has been studied.

The dynamic behaviors of the system are affected by modifying the frequency and amplitude parameters, according to numerical simulations. It should be noted as the existence of stochastic solutions is mostly determined by the parameter estimation and subsequently, by the specific nonlinear properties of the medium. Dynamical observations and the modified Sardar sub-equation technique have both been shown to be useful additional mathematical tools for the development of exact solutions and modified variational iteration technique for approximate solutions to their qualitative examination in NLEEs. Engineering, nonlinear sciences and mathematical physics are some of these disciplines.

## Conclusions

In a wide range of physical phenomena, such as nonlinear optics, optical communication systems, and plasma-based solutions, this work emphasizes the importance and applicability of the SNLSM in (1+1)-dimension with random potential. Our work offers fresh knowledge about the model’s traits and behavior despite the model having been widely studied in the literature. There have developed numerous stochastic soliton solutions, such as dark, hyperbolic, singular, plane wave, trigonometric, and periodic ones. The modified VI method also offers a powerful and flexible framework for studying nonlinear evolution issues. The solution and semi-analytical solutions found can help improve numerical simulation accuracy and help develop more reliable and effective models for a variety of real-world applications. The phase plane and time series analysis of the proposed model are also investigated. The novel solutions identified in this study show promise for application in a number of industries. In contrast to optical solitons and optical fiber communication systems, which may be modeled using periodic and plane wave solutions, optical pulse compression approaches, for example, may be constructed utilizing single stochastic solutions. The systematic and statistical framework of this method can help in the development of more precise and dependable numerical methods for solving difficult nonlinear evolution equations. Additionally, to demonstrate the graphical depiction of a few wave patterns with various investigated system features and to verify the accuracy of our results, we employed the Mathematica and Matlab software. When compared to those obtained by using standard methods, the stochastic solutions we describe are innovative. Future conversations in the nonlinear physical sciences will be stimulated and encouraged by the accomplishments of this work. The calculations also show us the value of the approaches for more broadly locating the precise stochastic solutions.
